# A Novel Detection and Classification Framework for Diagnosing of Cerebral Microbleeds Using Transformer and Language

**DOI:** 10.3390/bioengineering11100993

**Published:** 2024-09-30

**Authors:** Cong Chen, Lin-Lin Zhao, Qin Lang, Yun Xu

**Affiliations:** 1 School of Clinical Medicine, College of Medicine, Nanjing Medical University, Nanjing 211166, China; 2 Department of Neurology, The First Affiliated Hospital of Nanjing Medical University, Nanjing 210029, China; 3 Department of Computer Science and Technology, Shanghai University, 99 Shangda Road, Baoshan District, Shanghai 200444, China; 4 Department of Neurology, Nanjing Drum Tower Hospital, Affiliated Hospital of Medical School, Nanjing University, 321 Zhongshan Road, Nanjing 210008, China

**Keywords:** cerebral microbleeds, convolutional neural network, multimodal, detection and classification, language–vision

## Abstract

The detection of Cerebral Microbleeds (CMBs) is crucial for diagnosing cerebral small vessel disease. However, due to the small size and subtle appearance of CMBs in susceptibility-weighted imaging (SWI), manual detection is both time-consuming and labor-intensive. Meanwhile, the presence of similar-looking features in SWI images demands significant expertise from clinicians, further complicating this process. Recently, there has been a significant advancement in automated detection of CMBs using a Convolutional Neural Network (CNN) structure, aiming at enhancing diagnostic efficiency for neurologists. However, existing methods still show discrepancies when compared to the actual clinical diagnostic process. To bridge this gap, we introduce a novel multimodal detection and classification framework for CMBs’ diagnosis, termed MM-UniCMBs. This framework includes a light-weight detection model and a multi-modal classification network. Specifically, we proposed a new CMBs detection network, CMBs-YOLO, designed to capture the salient features of CMBs in SWI images. Additionally, we design an innovative language–vision classification network, CMBsFormer (CF), which integrates patient textual descriptions—such as gender, age, and medical history—with image data. The MM-UniCMBs framework is designed to closely align with the diagnostic workflow of clinicians, offering greater interpretability and flexibility compared to existing methods. Extensive experimental results show that MM-UniCMBs achieves a sensitivity of 94% in CMBs’ classification and can process a patient’s data within 5 s.

## 1. Introduction

Cerebral Micro Bleeds (CMBs) are diminutive hemosiderin deposits, a consequence of cerebral microvascular lesions in the brain parenchyma. These lesions, which appear as small, round, homogeneous, and hypointense areas, are detectable through Magnetic Resonance Imaging (MRI), particularly using T2-gradient-recalled echo (T2*-GRE) and susceptibility-weighted imaging (SWI) [1]. The detection of CMBs is a critical step in diagnosing and managing cerebral small-vessel disease (SVD) and is integral to identifying severe neurological conditions such as ischemic stroke, traumatic brain injury, and Alzheimer’s disease [2]. The integration of deep learning with disease diagnosis has become a major focus in medical image recognition tasks. Recently, due to the high costs of resource allocation and sample annotation in deep learning methodologies, researchers have explored Spiking Neural Networks (SNNs) [3,4] for medical applications. SNNs bridge the gap between neuroscience and machine learning by employing biologically inspired neuron models for computational tasks. For instance, Turkson [5] developed an innovative SNN pipeline to classify Alzheimer’s disease (AD) into three binary tasks: AD versus healthy controls (NC), AD versus Mild Cognitive Impairment (MCI), and NC versus MCI, using MRI scans. Additionally, ref. [6] proposed an optimized tumor segmentation approach that integrates a deep spiking neural network (DSNN) with a conditional random field structure to improve segmentation accuracy. To mitigate overfitting in classification models, a deep convolutional spiking neural network (DCSNN) [7] has been introduced for lung disease detection using chest X-ray images. These advancements underscore the growing interest in using deep learning techniques for disease diagnosis.

Due to the small sizes of CMBs in MR images, typically ranging from 2 mm to 10 mm in diameter, CMBs are susceptible to interference from other similar targets, such as calcifications and veins. As a result, accurately identifying genuine CMBs in SWI images poses a significant challenge for clinical practitioners. To address this issue, various automatic detection methods [8] have been proposed to assist clinical physicians in detecting CMBs. These methodologies can be classified into one-stage [9] and two-stage approaches [10]. One-stage methods offer higher integration and faster inference speeds, whereas two-stage approaches can provide superior performance by separating detection from classification tasks. Given the stringent accuracy requirements in CMBs’ detection and classification, current research has focused on the two-stage architecture. In this framework, CMBs’ candidate regions, which include both true CMBs and their mimics, are identified in the detection phase and then classified in the subsequent classification stage [11]. The two-stage pipeline has proven effective in reducing false positives. However, it still presents several unique challenges: (1) balancing performance with speed; (2) reducing annotation costs for training samples; (3) accurately detecting the small and weakly featured CMBs; and (4) distinguishing genuine CMBs from similar-looking targets.

In this paper, we present a decoupled two-stage framework for CMBs’ detection and classification, named MM-UniCMBs, which employs a front-light (detection stage) and back-heavy (classification stage) design. The detection stage is optimized for speed, focusing on rapid processing, while the classification stage is designed to prioritize high accuracy. This decoupled pipeline effectively balances detection speed with classification accuracy, addressing the first challenge. During the labeling process, we observed that classification requires less labeling effort compared to object detection, largely due to the labor-intensive nature of bounding box annotations in the latter. Leveraging this observation, we employ distinct labeling strategies for each phase. In the detection stage, preliminary assessments are made to identify images with potential CMB regions, which does not demand extensive clinical expertise and can involve less experienced intern doctors. In contrast, the classification stage requires clinicians with substantial expertise to make definitive judgments on whether the identified regions contain true CMBs. This bifurcated approach reduces the need for expert involvement in the initial labeling phase, effectively addressing the challenge of annotation costs.

In response to the third challenge of detecting small and weakly featured CMBs, we conducted an analysis of CMB features in SWI images and developed a lightweight 2D Convolutional Neural Network (CNN)-based detection model, named CMBs-YOLO. To strengthen the weak features of CMBs, a novel CMBs-Multi Head Attention (CMBs-MHA) module is proposed, specifically designed to accentuate the distinct disparities between CMB targets and their surrounding background. Simultaneously, to bolster the robustness of modeling small features, we incorporated the Normalized Wasserstein Distance (NWD) [12] method as a supplementary loss, complementing the conventional Intersection over Union (IoU)-based [13] approach.

Faced with the final challenge, we developed an innovative multimodal classification network, named CMBsFormer (CF). This network stands out by integrating text language descriptions with a transformer-based architecture, incorporating the clinical knowledge into the classification modeling process. Our CF framework encapsulates two critical aspects of clinical diagnosis: looking (patient’s MRI images) and asking (patient’s age, gender, past medical history, lifestyle habits, etc.), thus aligning the modeling process more closely with the diagnostic procedures used by clinicians. Meanwhile, the adoption of the transformer structure can achieve an advancement compared to the conventional CNN-based methods, as this structure excels in feature extraction and demonstrates superior proficiency in integrating textual embedding information. Additionally, the proposed CF framework exhibits strong adaptability and flexibility across various configurations.

In summary, our contributions can be summarized as follows:

(1) We introduce a decoupled two-stage framework named MM-UniCMBs, characterized by the front-light and back-heavy architecture;
(2) We propose a novel lightweight CMBs detection network called CMBs-YOLO, which incorporates the CMBs-MHA module and NWD loss;
(3) An innovative multimodal classification network named CMBsFormer is proposed by integrating text language descriptions with a transformer-based architecture;
(4) The extensive experimental results demonstrate that our proposed framework can achieve satisfactory performance in both the CMB detection and classification tasks.

The subsequent chapters in this paper are organized as follows. Section 2 provides an overall review of CMBs’ detection and classification. In Section 3, we present a description of our CMBs dataset, along with the details pertaining to our detection and classification models. The comparative experimental results and discussions are presented in Section 4. In Section 5, we provide a summary and ablation studies for the field of automated detection and classification of CMBs. The discussion and conclusion are present in Section 6. Finally, the detailed experimental results of our method are presented in the Appendix A, Appendix B and Appendix C.

## 2. Related Work

In the field of CMB detection and classification, early studies relied on manually predefined features such as intensity thresholds, area size, and other traditional image processing techniques [14,15,16,17,18,19,20]. Dou et al. [21] modeled the detection of CMBs through a three-stage process: initial candidates screening based on intensity values, extraction of compact 3D hierarchical features via a stacked convolutional Independent Subspace Analysis (ISA) network, and elimination of false positives using a Support Vector Machine (SVM) classifier, which leveraged the representation features learned from ISA. Another contribution in [10] employed three distinct machine learning classifiers, SVM, Quadratic Discriminant Analysis (QDA), and an ensemble classifier, to detect and classify CMBs. Fazlollahi et al. [22] introduced a multi-scale Laplacian of Gaussian technique. This technique was designed to detect the potential CMB candidates, along with their corresponding bounding boxes, followed by a cascade of binary random forests to refine detection accuracy. Bian et al. [23] utilized the 2D fast radial symmetry transform for initial detection of candidate CMBs and subsequently applied 3D region growing to eliminate false positives. In another study, Fazlollahi et al. [24] implemented a cascade of random forest classifiers trained on robust radon-based features, particularly addressing the challenge posed by unbalanced sample distribution. Morrison et al. [25] incorporated a 2D fast radial symmetry transform and handcrafted features to automatically detect CMBs with high sensitivity. While these early methodologies laid the groundwork for the evolution of CMB detection and classification, they struggled to achieve satisfactory performance due to the limited feature-learning capabilities and inadequate representational power of manually extracted features.

The advent of deep learning algorithms has significantly revolutionized the field of CMB detection and classification [26,27,28,29,30,31,32,33]. In [34], the researchers employed a fully automated two-stage integrated deep learning strategy by combing a regional-based method called You Only Look Once (YOLO) for initial CMB candidate detection, followed by a 3D-CNN classification stage to reduce false positives. Fang et al. [35] focused on generating heatmaps of CMBs by proposing a 2.5D CNN model to establish a priori knowledge base, addressing the redundant computation in 3D-CNN and the loss of spatial information in 2D-CNN. A novel CMB classification method was proposed in [36], which integrated a deep CNN model with randomized neural networks, a Random Vector Functional Link network (RVFL), and an Extreme Learning Machine (ELM). This combination aimed to balance the image feature learning capabilities with the training speed. Koschmieder et al. [37] developed distinct approaches for the detection of CMBs in traumatic brain injury (TBI) cases, including a classification method (Patch-CNN) and two segmentation methods (U-Net). Ferlin et al. [9] proposed a machine learning approach based on a 2D Faster R-CNN network, achieving a balance between sensitivity and precision. Stanley et al. [38] introduced a novel feature extraction technique for CMB detection, using a CNN extractor combined with Long Short-Term Memory (LSTM) to classify selected Regions of Interest (ROI) as CMBs or non-CMBs. A 3D patch-based deep residual network was implemented in [39,40], specifically designed to differentiate true CMBs from their mimics in SWI images. Compared to traditional machine learning methods, the CNN-based approaches have achieved a significant performance improvement in both detection and classification. However, these methods still have limitations due to their sole reliance on image information. This one-dimensional focus creates a substantial gap between these methods and the diagnostic processes employed by clinical doctors, resulting in a bottleneck in current research.

The emergence of multimodal methods in the medical domain, particularly language–vision models, has demonstrated a remarkable capability to extract and integrate representations from both images and text descriptions [41]. One notable example is the Language meets Vision Transformer (LViT) [42], a language–vision medical image segmentation architecture that utilizes a hybrid CNN–Transformer structure for lung organ segmentation. Liu et al. [43] leveraged the Contrastive Language–Image Pretraining (CLIP) [44] method to effectively integrate text embeddings with medical image segmentation models. Furthermore, Bhalodia et al. [45] introduced a novel pneumonia detection technique based on attention mechanisms, utilizing text information extracted from medical reports. These language–vision methods have attained considerable success across various medical application fields. However, to the best of our knowledge, these approaches have not been employed in the domain of CMBs’ detection or classification. In this article, we will explore the applicability of multimodal methods in the task of CMB classification, focusing on the integration of MRI images with patient descriptors.

## 3. Materials and Methods

### 3.1. Data Acquisition

In this study, we collected a dataset of CMBs involving 600 patients from Nanjing Medical University’s Affiliated Jiangsu Province Hospital. The data collection spanned from 1 January 2022 to 1 October 2023. All MRI scans were acquired on the 3.0T GE scanner (Discovery MR750, GE Medical Systems, Milwaukee, WI, USA). Each patient’s data included 130 SWI slices and 130 phase slices, all rendered at a resolution of 768 × 768 pixels. Alongside these images, we incorporated the patient information such as age, gender, past medical history, and lifestyle habits. It is important to note that the inclusion of past medical history and lifestyle habits was applicable only to a limited subset of patients. The diagnostic evaluations of all patients were reviewed by clinical physicians. The identification and annotation of CMB targets in these patients were conducted by neurologists with 15 years of clinical experience. In the case of labeling discrepancies, another senior neuroradiologist was consulted, with the final determination made based on a consensus among these experts. To maintain consistency in analysis, the grayscale values of each SWI and phase image were normalized to a range of 0–255 and then converted into three-channel format. Additionally, we incorporated a supplementary public dataset of CMBs from the VALDO 2021 challenge [46]. For clarity, we refer to these datasets as CMBs-Private and CMBs-Public, respectively. Table 1 and Table 2 provide detailed information on the data partitioning and distribution for training and testing purposes.

### 3.2. Methods

**Figure 1:**
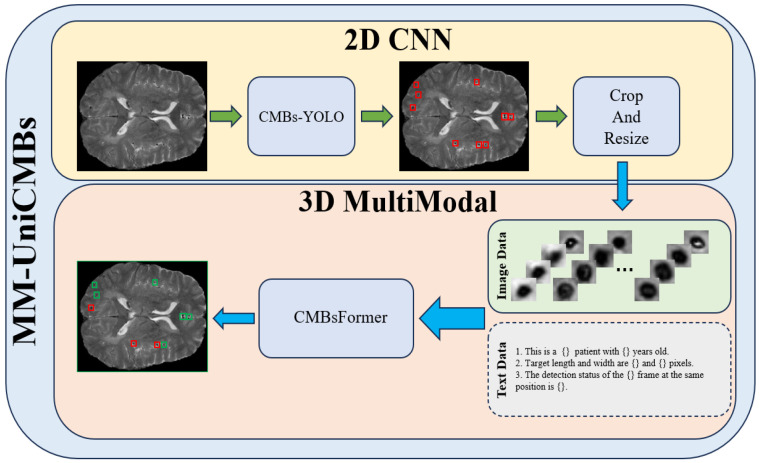
The architecture of MM-UniCMBs.

In this section, we will discuss the implementation details of our MM-UniCMBs. The structure of MM-UniCMBs is illustrated in Figure 1. During the initial detection stage, only SWI images are processed to identify potential candidate regions of CMBs. Subsequently, the classification model takes SWI data and text descriptions as inputs to eliminate false positives.

#### 3.2.1. CMBs-YOLO

**Figure 2:**
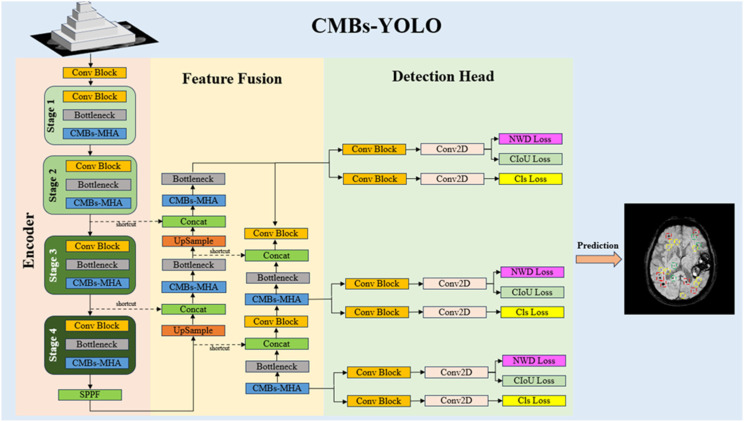
The structure of CMBs-YOLO.

At present, prevalent object detection architectures can be classified into CNN-based methods [47] and transformer-based methods [48]. Compared to the transformer structures, CNNs can offer a more favorable balance between performance and speed. An excellent example of CNN methods is Ultralytics YOLOv8, a universal state-of-the-art (SOTA) model in object detection tasks, which builds upon the achievements of earlier YOLO iterations [49]. YOLOv8, recognized for its swiftness and high performance, is an ideal choice for our CMB detection task. However, despite its strengths, the standard YOLOv8 structure still has limitations, particularly in terms of feature modeling capability for weak features and the design of its loss function for small targets. To address these issues, we proposed a novel CMB detection network, termed CMBs-YOLO, as illustrated in Figure 2. By incorporating CMBs-MHA modules in both the feature extraction and fusion stages, the feature learning ability for weak features can be enhanced. Regarding to bounding box regression, we have refined the existing Complete Intersection over Union (CIoU) loss with an additional NWD loss, aiming to augment the stability of the detection network, particularly for small targets.

**Figure 3:**
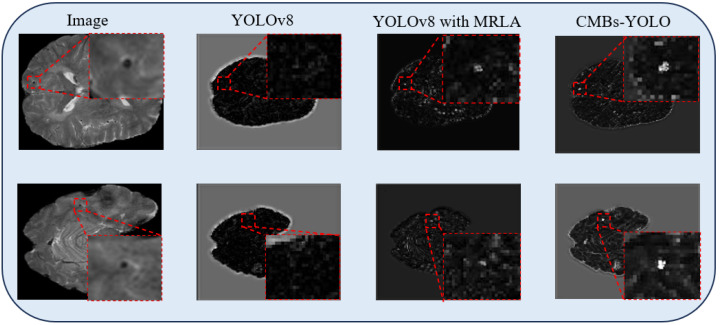
Feature responses of CMBs in MRI.

Regions containing CMB targets exhibit distinguishable differences from the surrounding background areas in SWI. However, this critical characteristic has been overlooked in existing research on the detection of CMBs, leading to a decline in performance. As depicted in Figure 3, the standard YOLOv8 structure struggles to model this difference, leading to missed detections. We should emphasize that incorporating Multi-Head Recurrent Layer Attention (MRLA) [50] in the encoding stage can enhance the interaction ability of layers in the CNN structure, thereby intensifying the network’s attention to diverse features. Integrating MRLA into the YOLOv8 framework, as demonstrated in Figure 3, has indeed improved the detection of CMB targets. However, it is also important to note that the original structure of MRLA was not specifically tailored to accentuate the distinct features of CMB targets. As a result, although the MRLA module brings an improvement in the detection of CMBs, its capability in modeling saliency features remains limited.

**Figure 4:**
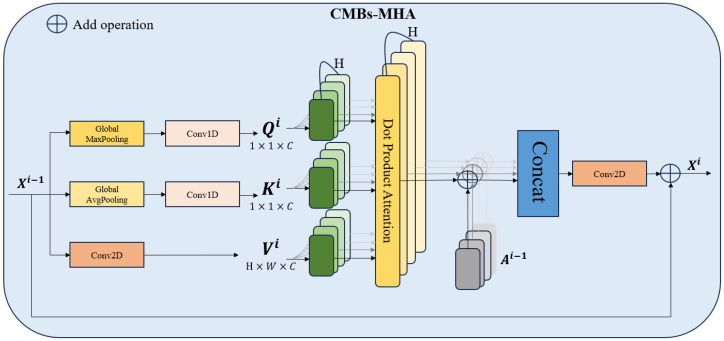
The overview of the CMBs-MHA module.

To enhance the representation capabilities across various encoding layers and amplify the distinct responses of CMB targets against the background, we introduce the CMBs-MHA module; its structure is shown in Figure 4. The key innovation of CMBs-MHA lies in its utilization of the global max pooling operation to generate query vectors instead of global average pooling, which can help in extracting salient regions from the entire feature map. Meanwhile, it employs a global average pooling operation to create key-value vectors, constructing a background board to model local features. Subsequently, a multi-head attention block is applied on these queries and key vectors to build the saliency relationships among CMBs’ features and their surrounding background. As illustrated in Figure 3, the incorporation of the CMBs-MHA module has significantly enhanced the responses of CMB features on the feature map. Additionally, our CMBs-MHA module is designed for seamless integration as a plugin into any CNN-based detection framework.

**Figure 5:**
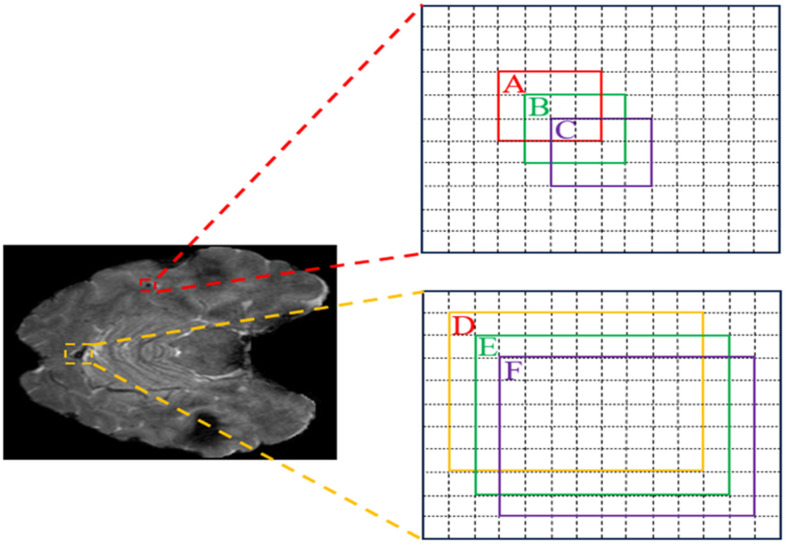
The relationship between the predicted boxes and ground truth boxes.

Another frequently overlooked aspect in the CMBs detection task relates to the design of the bounding box regression. The small size of CMB targets, typically ranging from 2 mm to 10 mm, corresponds to an occupation of 4–32 pixels on each SWI image. However, the prevalent design of box regression loss, which is based on the IoU theory, has been found to be less efficient for these tiny targets, as indicated in [12]. To illustrate this point, we provide a visual representation of the relationship between the predicted bounding boxes and ground truth boxes in Figure 5, including both small and standard sizes. In this context, A and D denote the ground truth boxes, while B, C, E, and F represent the predicted boxes, with each box being separated by one pixel. This visual representation can help us to understand the challenges associated with solely relying on IoU-based loss functions for the detection of CMB targets.

In Table 3, we present a comparative analysis using different loss functions, focusing on two distinct target sizes: small and standard. Two key issues arise when applying IoU-based methods for the detection of CMBs. Firstly, even a one-pixel discrepancy, as observed between (A, B) and (D, E), leads to a substantial variation in calculated IoU values, with differences reaching up to threefold. Secondly, a size change in a small-sized CMB target from B to C results in an IoU reduction of 0.23, while a similar size change in a normal-sized CMB target from E to F only alters the IoU by 0.07. These observations underscore the limitations of IoU-based methods in processing the small targets. The significant fluctuation in IoU values due to minor positional changes, particularly for small-sized CMB targets, highlights the necessity of introducing an additional loss in the detection of CMBs. To this end, we have integrated the NWD loss into our CMBs-YOLO network.

Given a predicted bounding box $A=({Px}_{1},Py_{1},Px_{2},{Py}_{2})$ and a ground truth box $B=(Gx_{1},Gy_{1},Gx_{2},{Gy}_{2})$, the NWD loss can be computed as follows:

(1) $$WD\left(A,B\right)=e^{-\frac{\sqrt{r_{dis}(A,B)+w_{dis}\left(A,B\right)}}{C}}$$

(2) $$r_{dis}\left(A,B\right)=\left(\frac{{Px}_{1}+Px_{2}}{2}-\frac{Gx_{1}+Gx_{2}}{2}\right)\times \left(\frac{Py_{1}+{Py}_{2}}{2}-\frac{Gy_{1}+{Gy}_{2}}{2}\right)+\delta$$

(3) $$w_{dis}\left(A,B\right)=\frac{{\left(\left(Px_{2}-{Px}_{1}+\delta \right)-\left(Gx_{2}-Gx_{1}+\delta \right)\right)}^{2}+{\left(\left({Py}_{2}-Py_{1}+\delta \right)-\left({Gy}_{2}-Gy_{1}+\delta \right)\right)}^{2}}{4}$$

where *C* and *δ* are constants with values set to 12.8 and 1 × 10^−7^ in our study, respectively. Examination of the results in Table 3 reveals that the NWD can enhance the robustness of small targets in comparison to IoU-based methods.

In the detection stage, we addressed the performance bottlenecks in the detection of CMBs by focusing on two aspects: feature modeling and loss function design. The integration of the CMBs-MHA module during the encoding stage enhances the network’s ability to capture salient responses of CMB targets against the background. Additionally, the implementation of the NWD is essential in improving the training stability of the network, particularly for small targets. The architecture of our CMBs-YOLO is designed to enhance the adaptability and effectiveness of the YOLO framework for the specific demands of detecting CMBs.

#### 3.2.2. CMBsFormer

Following the detection phase, numerous candidate CMB regions can be identified, however, containing a large number of false positives. Unlike other typical image classification tasks, the classification process for CMBs requires integrating multiple slice images. Two primary approaches have been developed for the design of CMB classification networks based on the processing method of input images. The first approach entails processing multiple cropped slice images, which are either stitched together or averaged, to convert them into 2D input formats. Following this transformation, 2D convolutional networks are employed to classify true CMB targets [34]. This approach struggles to achieve satisfactory classification results due to the loss of spatial information in adjacent slice images. To address this, contemporary CMB classification approaches emphasize the use of 3D-CNN structures with 3D image data to enhance the network’s modeling ability. Additionally, some researchers have integrated manually designed features, such as texture or geometric information, to expand the feature set of the classification network [52]. Nevertheless, the above methods still face two notable challenges in classifying CMBs. Firstly, CNN-based methods have limited capacity to differentiate between CMBs and their mimics. Secondly, manually engineered features, which are typically specific to certain datasets, exhibit poor interpretability and flexibility. Meanwhile, the poorly designed manual features can degrade network performance.

**Figure 6:**
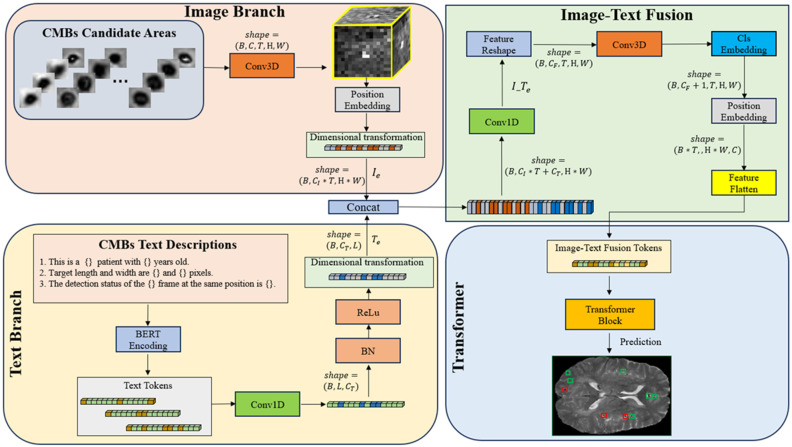
The architecture of CMBsFormer.

In this paper, we explore the inter-relationship between patient’s textual information and SWI data, presenting a novel multimodal CMB classification network. The architecture of CF is visually depicted in Figure 6. The CF architecture consists of four components: the image branch, the text branch, the image–text fusion module, and the transformer classification module. The architecture is designed to accommodate both multimodal (image and text) and single-modal (image) inputs, offering flexibility across different configurations. For clarity, we focus on demonstrating the multimodal input configuration.

In our CF framework, the image branch is designed to extract feature information from 3D SWI and phase images. Assuming the input images are $I=\{S_{i-1},\;S_{i},S_{i+1},P_{i}\}\in R^{B\times C\times T\times H\times W}$, the process of feature extraction is articulated through the following equations:

(4) $$I_{conv\_1}=GELU(BN3D(Conv3D(I)))$$

(5) $$I_{conv\_2}=BN3D(Conv3D(I_{conv\_1}))$$

(6) $$I_{pos}=I_{conv\_2}+pos\_embedding(I_{conv\_2})$$

(7) $$I_{e}=reshape(I_{pos}){\in R}^{B\times (C\times T)\times (\frac{H}{4}\times \frac{W}{4})}$$

where $S_{i}$ and $P_{i}$ represent the SWI and phase images. Conv3D, BN3D, and GELU refer to 3D convolutional, 3D batch normalization, and the GELU activation functions, respectively. *B* denotes the batch size, and *C*, *T*, *H*, and *W* represent the embedding channels, input image slices, height, and width, correspondingly. Through two convolution operations, the width and height of the input images are reduced from 64 to 16, and the number of channels is increased from 3 to 512. Position embedding data are added after the convolution operations to model the positional information of different image patches. Finally, the 3D image data are reshaped into a 1D structure by amalgamating channels and slices as well as width and height, obtaining the image branch output $I_{e}\in R^{B\times (C\times T)\times (\frac{H}{4}\times \frac{W}{4})}$.

By analyzing the clinical diagnostic process for CMBs, we observed that their occurrence is closely associated with several factors, including a patient’s age, gender, and lifestyle habits. Additionally, the patient’s medical history—encompassing conditions such as Cerebral Autosomal Dominant Arteriopathy with Sub-cortical Infarcts and Leukoencephalopathy (CADASIL), diabetes, hypertension, and coronary heart disease—plays a critical role in diagnosing CMBs. Currently, no methods are available that incorporate such detailed patient information, leading to a significant gap between model-based processing and clinical diagnostic procedures. In our research, we treat the process of inquiring about patient’s information as a method for modeling patient text data. Of course, due to privacy concerns and the sensitive nature of information related to patients’ lifestyle habits and medical histories, these elements were excluded from our analysis. Nevertheless, we will discuss these aspects in some specific cases in the experimental section. Table 4 presents the structure of our text prompts and their corresponding descriptions, illustrating how these elements were integrated into our research.

Assume the text descriptions in our study are represented as $T=\{T_{1},\;T_{2},\ldots T_{n}\}$, where T can be replaced or extended to other text statements, such as the patient’s lifestyle habits and past medical history. To process the textual data, we utilize a pretraining model named Bidirectional Encoder Representations from Transformers (BERT) [53]. This model can transform each individual text statement into a 768-dimensional word vector. Subsequently, these individual word vectors are then aggregated to form an n-dimensional tensor. Following this, a permute operation is applied to rearrange this stacked tensor, obtaining the text branch output $T_{e}\in R^{B\times 768\times n}$.

We execute the fusion of image–text features and the classification of CMBs through the following steps. In the first step, both $I_{e}$ and $T_{e}$ are transformed into 1D data structures and then concatenated together along the channel dimension. A subsequent 1D convolution operation is performed, yielding the image–text fusion features denoted as ${I\_T}_{e}$, which will be converted into a data structure of the form (*B*, *C*, *T*, *H*, *W*). The second step involves the use of 3D convolution to further process the fusion information derived from both image and text dimensions. This step not only models the combined information but also integrates classification and positional encoding vectors for the following classification stage. The third step involves flattening the 3D tensor into a 1D format, a procedure for compatibility with the following transformer structure. Finally, a multi-head transformer block is utilized to extract features and classify CMBs. In scenarios where the model is provided solely with image inputs, our CF framework can adapt accordingly. It circumvents the feature fusion process, and instead, the results of the 3D convolution are directly channeled into the transformer module for the final classification stage.

By integrating 3D image data and text information into a unified network, our CF framework addresses several key challenges. Firstly, by replacing the traditional 3D-CNN structure with a transformer architecture, CF tackles the problem of feature extraction capability for both CMBs and their mimics. This architectural shift can enhance the network’s ability to discern and extract features more accurately. Secondly, CF incorporates text encoding as an alternative approach to manually designed features, allowing the model to leverage the specialized knowledge of clinical experts to enrich its information base while also improving interpretability. Moreover, the text prompts used in our study are designed to be dataset independent, offering flexibility for incorporating other relevant prompts. Finally, the text embedding structure demonstrates superior compatibility with the transformer architecture compared to CNN-based approaches.

## 4. Results

### 4.1. Evaluation Metrics

In this paper, we introduced a two-stage model for the detection and classification of CMBs. To evaluate the model’s performance, we employ different evaluation metrics at each stage. In the detection phase, we utilize precision (P), recall (R), and mean Average Precision (mAP) [48] as evaluation metrics, all based on the IoU method. In the classification stage, we introduced additional evaluation metrics, including sensitivity (also known as the true-positive rate, TPR) [54], specificity (also known as the true-negative rate, TNR) [54], and precision (P) [54]. To assess the model’s accuracy at the patient level, we incorporated FPavg [54] and $P_{patient}$ as supplementary evaluation metrics. Here, FPavg represents the average number of false-positive predictions per patient, providing insights into the model’s tendency towards over-diagnosis. $P_{patient}$ measures the model’s overall precision at the patient level, reflecting its performance in two key aspects: (1) error-judgment, where the model erroneously identifies a patient as having CMBs when they do not, and (2) missing-judgment, where the model fails to detect the presence of CMBs in a patient who has them. The calculations for these metrics in the classification stage are elaborated upon as follows:

(8) $$Sensitivity=\frac{TP}{TP+FN}$$

(9) $$Specificity=\frac{TN}{TN+FP}$$

(10) $$Precision=\frac{TP}{TP+FP}$$

(11) $$FPavg=\frac{FP}{n}$$

(12) $$P_{patient}=\frac{\begin{array}{c} \sum_{i=0}^{n}{patient}_{i}=\left\{\begin{array}{c} 1,if\sum_{j=0}^{S}{TP}_{j}>0\;and\;if{\;patient}_{i}\;has\;\;CMBs\\ 0,otherwise \end{array}\right.\\ +\\ \sum_{i=0}^{n}{patient}_{i}=\left\{\begin{array}{c} 1,if\sum_{j=0}^{S}{TP}_{j}=0\;and\;if{\;patient}_{i}\;has\;not\;CMBs\\ 0,otherwise \end{array}\right. \end{array}}{n}$$

where TP is the number of correctly classified positive samples, TN is the number of correctly classified negative samples, FP is the number of false positives, and FN is the number of false negatives (missed CMBs), n is the number of patients in the test set, and S is the number of SWI slice images per patient; its value is 130 in this paper. ${TP}_{j}$ represents the number of true CMBs regions on the j-th slice image.

### 4.2. Experiment Setup

The hardware and software utilized in this study are as follows. An Intel Xeon Gold 6330 CPU operating at 2.0 GHz, 64GB RAM, and a NVIDIA TITAN RTX with 24GB GPU memory. The software environment includes Python 3.8.5, CUDA 11.1, and cuDNN 8.9.2 for GPU-accelerated libraries.

### 4.3. Evaluation of Model Performance

#### 4.3.1. Detection Performance of CMBs

In this section, we evaluate the performance of various detection models, focusing on three distinct research directions: CNN-based detection methods, transformer-based methods, and the YOLO series. Our objective is to provide a comprehensive analysis of the strengths and limitations of these different approaches in the context of detecting CMBs. To ensure consistency in our evaluation, all models were trained using input images with a resolution of 512×512. Additionally, data augmentation techniques, including *RandomResize*, *RandomFlip*, and *RandomCrop*, were applied during the training phase for the CMBs-Public dataset. The training configuration details are outlined in Table 5, and the comparative results are presented in Table 6 and Table 7.

The comparative results in Table 6 reveal that our CMBs-YOLO model outperforms other detection methods, with CNN-based methods showing the lowest performance. A notable trend observed across all models is the higher achievement in P metrics compared to R. It indicates a prevalent challenge of missing detections, predominantly attributed to the subtle features of CMB targets. Our CMBs-YOLO model, scaled at X6, achieves the highest precision and recall rates, exhibiting an approximately 5% improvement over transformer-based models. This improvement underscores the efficacy of the CMBs-MHA module and the NWD loss functions implemented in our study. Meanwhile, our proposed method does not entail a substantial increase in learnable parameters and inference time. For example, the smallest variant of the CMBs-YOLO (N) model increases only an additional 0.46 million parameters but achieves a 9% improvement in the ${mAP}_{50-95}$ metric compared to the YOLOv8 model, demonstrating a favorable balance between performance and speed.

Analysis of the CMBs-Public dataset, presented in Table 7, reveals a notable decline in the R metric across all evaluated models. This decrease can be attributed to two factors: first, the limited number of labeled samples of CMBs in the CMBs-Public dataset impairs the models’ ability to effectively model features, leading to underfitting, as exemplified with the Dino method, which uses a transformer structure and experienced a substantial drop in the R index from 0.89 to 0.48. Second, the discrepancy in labeling strategies between the CMBs-Private and CMBs-Public datasets contributes to this decline. Our private dataset includes a broader range of similar targets labeled as CMBs, resulting in a higher number of labeled samples, while the public dataset follows a stricter definition. This difference, combined with the need for models to address both localization and classification of CMBs in a single stage on public datasets, increases network design complexity and limits performance.

In contrast, our study advocates for a decoupled design, where the localization and classification of CMBs are handled in separate stages. This design allows the detection model to focus on identifying candidate CMBs regions in the first stage, without the added complexity of distinguishing false positives, thereby improving the model’s performance, as demonstrated by the differences in results between the public and private datasets.

Additionally, it is noteworthy that the YOLO series detection methods exhibit less pronounced performance degradation on the CMBs-Public dataset compared to transformer-based models. For instance, the CMBs-YOLO (X6) method shows a reduction in the *P* and *R* metrics by 0.12 and 0.24, respectively. This suggests that YOLO detectors are better suited for scenarios with limited sample sizes, whereas transformer-based models often require a larger volume of labeled samples to fully leverage their modeling capabilities.

**Figure 7:**
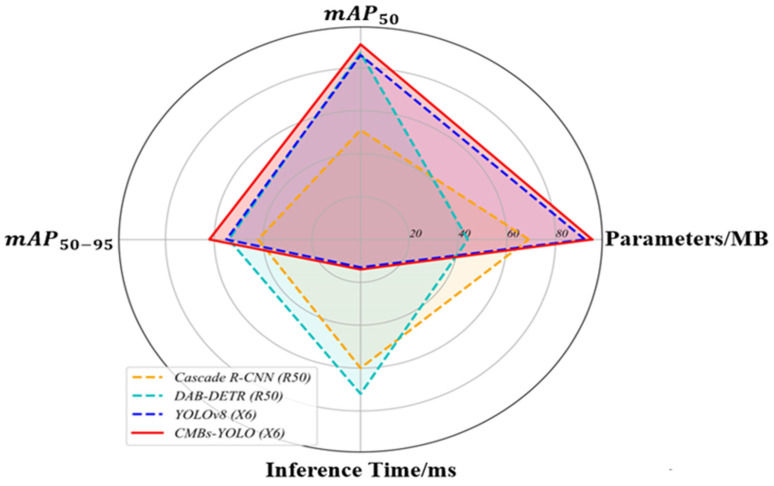
The evaluation of various models across different metrics in the CMBs-Private testing dataset.

**Figure 8:**
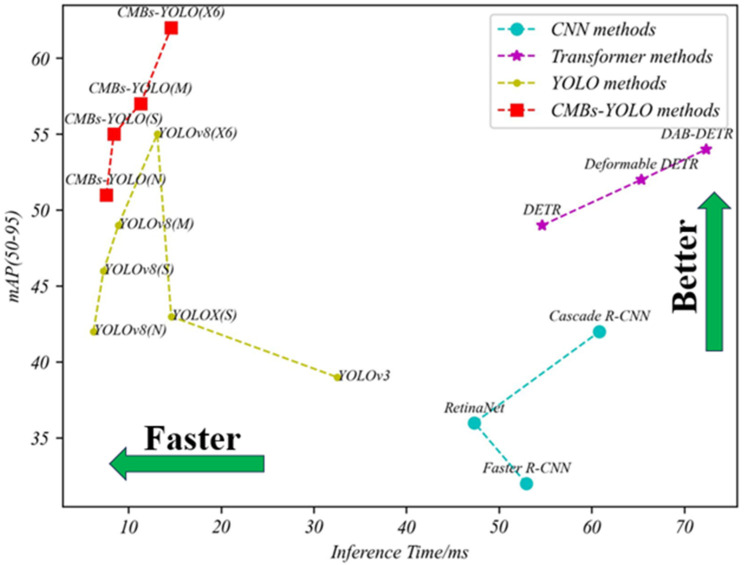
Comparing the trade-off between speed and performance among detection models in the CMBs-Private testing dataset.

**Figure 9:**
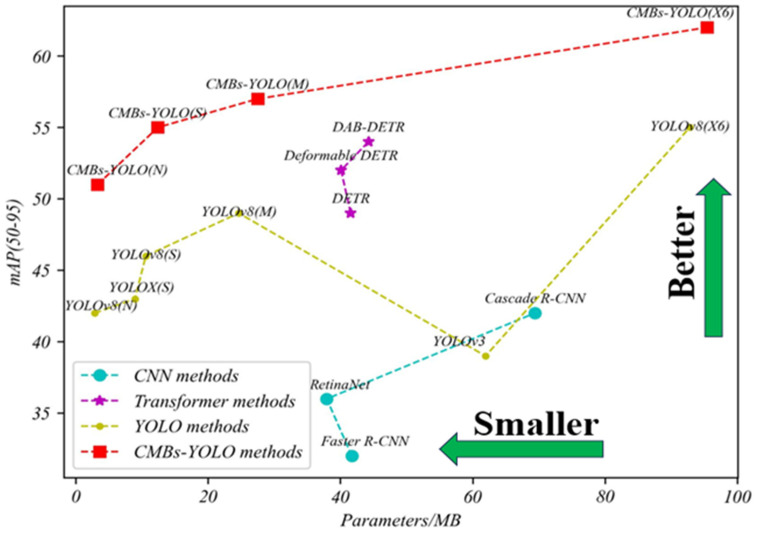
Comparing the trade-off between parameters and performance among different detection models in the CMBs-Private testing dataset.

Figure 7 illustrates the relationships between mAP indicators, inference speed, and parameters across different models, providing a clear visual understanding of performance dynamics. Additional comparisons regarding parameters and speed can be found in Figure 8 and Figure 9.

**Figure 10:**
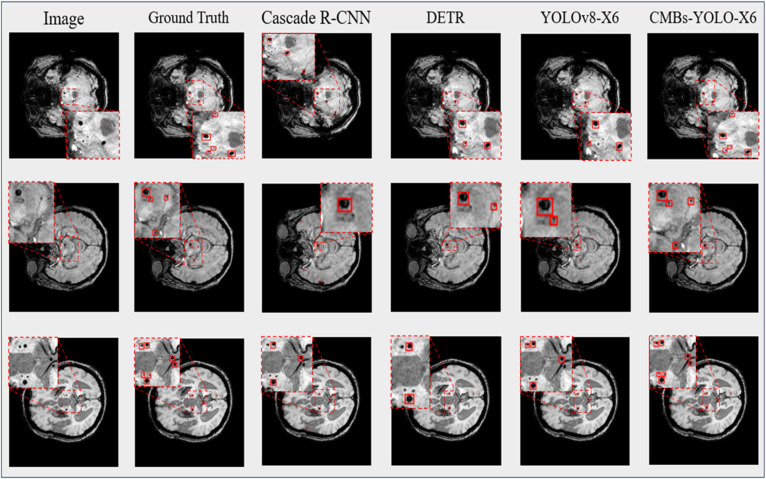
Visual results of detection models in CMBs-Private testing dataset.

In Figure 10, we present a partial visualization of results obtained from various detection models. Detailed detection results for our CMBs-YOLO model can be found in the Appendix A, Appendix B and Appendix C. It is evident that, except for our CMBs-YOLO, all other models exhibit issues of erroneous and missed detections. As these methods are general-purpose detection networks and are not specifically optimized for detecting CMBs, they demonstrate limited capabilities in identifying CMB targets.

#### 4.3.2. Classification Performance of CMBs

In the detection phase, numerous candidate CMB regions are identified, yet these regions contain a considerable number of false positives. Accurately classifying these regions is crucial for the automated diagnostic process. To ensure a fair evaluation of different classification models, this section assesses their performance within the context of 3D structures. Notably, transformer-based methods have not yet been explored in research on CMBs. To address this gap, we have incorporated the latest advancements from the video classification domain, specifically UniFormer v1 (UFv1) [67] and UniFormer v2 (UFv2) [68], into our comparative analysis. The performance comparison among different classification models is based on results obtained from our CMBs-YOLO (X6) model. Additionally, the methods discussed in this section utilize the CMBs-Private dataset for comparison.

Table 8 presents the results of various classification models, revealing that networks based on transformer structures generally outperform those utilizing CNN architectures. This observation suggests that transformer methods offer more advanced feature representation capabilities, particularly for handling 3D image data. However, CNN-based models retain an advantage in terms of parameters and inference speed. An intriguing observation from Table 8 is that the escalation in the number of parameters for transformer methods does not invariably lead to corresponding improvements in performance. For example, the UFv2 model demonstrated only a 2% enhancement in the P metric when its parameters increased from 108 M to 336 M. This phenomenon implies that once the feature extraction capacity of the transformer structure reaches a certain threshold, network complexity may no longer be the bottleneck for improving the accuracy of CMB classification.

**Figure 11:**
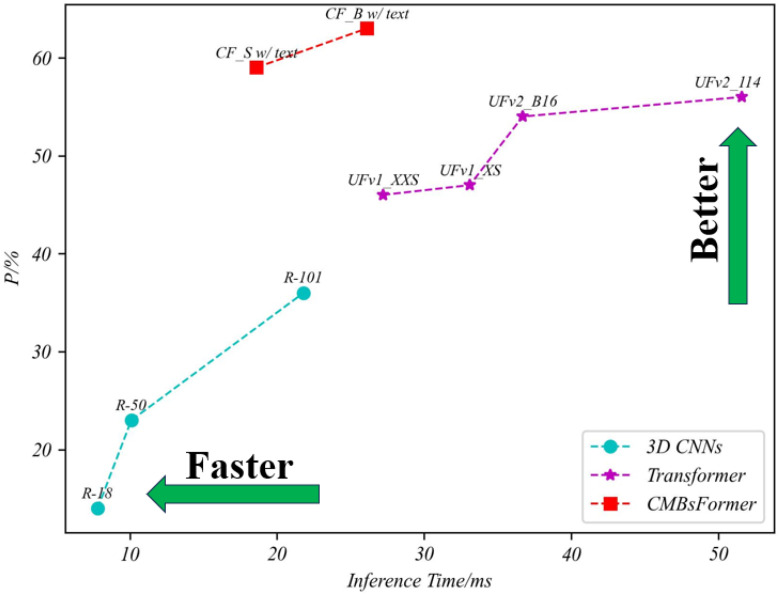
Comparing the trade-off between speed and performance in the CMBs-Private testing dataset.

**Figure 12:**
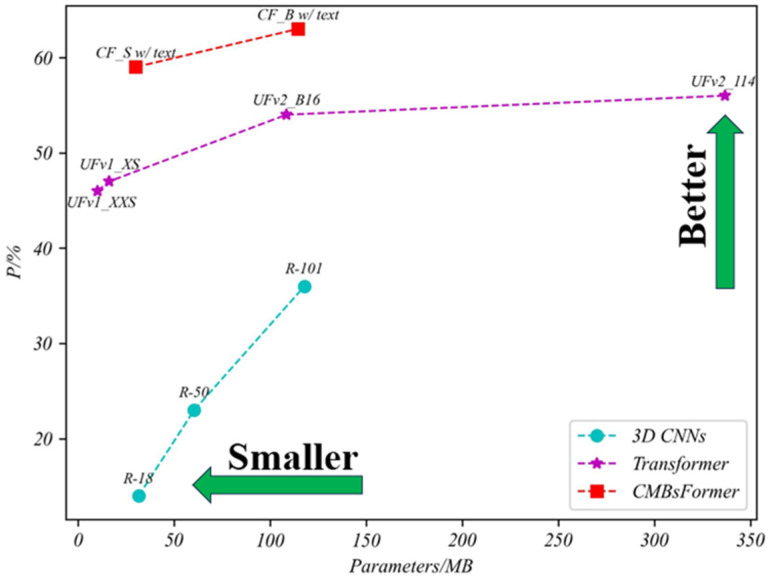
Comparing the trade-off between parameters and performance in the CMBs-Private testing dataset.

The integration of text description features into our CF model, alongside 3D image features, has significantly improved the accuracy of CMB classification. When comparing models with similar network complexities, such as UFv2_B16, CF_B without text, and CF_B with text, the CF_B with text configuration exhibited a notable performance enhancement. Specifically, it attained a 7% and 12% increase in the P metric compared to UFv2_B16 and CF_B without text models, respectively. This enhancement underscores the critical importance of incorporating text description content into the classification network, highlighting the value of the “asking” step in the clinician’s procedure. For a more intuitive and visual comparison, readers are directed to Figure 11 and Figure 12, which provide clear insights into the comparative strengths and weaknesses of each model.

In Table 9, we present partial visualization results obtained from various classification models. In this table, $S_{i-1}$, $S_{i}$, and $S_{i+1}$ represent the SWI images at slices *i* − 1, *i*, and *i* + 1, respectively, while $P_{i}$ represents the corresponding phase image. Notably, in the case of the first and last patients, only the CF_B model, which incorporates additional text information, accurately identified the regions as non-CMBs. Conversely, all other models incorrectly classified these regions as CMBs, due to the high similarity between interfering targets and CMBs. It is noteworthy that the first and last patients are inferred to be relatively young females based on gender and age. Models lacking patient-specific information often struggle with accurate classification in such cases. For a more comprehensive view of results from our method, please refer to the Appendix A, Appendix B and Appendix C.

#### 4.3.3. Total Performance of CMBs

In Section 4.3.1 and Section 4.3.2, our analysis focused on comparing the performance of different models in terms of detection and classification stages. In this section, we shift our attention to evaluate the performance of various model combinations at the patient level. Recognizing the vast array of possible combinations, we have selectively included only the top-performing model from each method category for reference. To guarantee a fair and balanced comparison, especially in terms of inference speed, we have standardized the number of potential CMB regions identified via different detection models to 100. The final total inference time metric can be computed as the average processing time across N patients, using the following formula: ${infer}_{time}=\frac{\sum_{i=0}^{N}{Num}_{image}\times D_{image}+100\times C_{image}}{N}$, where ${Num}_{image}$ represents the number of slice images for each patient taking a constant value of 130 in this paper. $D_{image}$ denotes the time taken by the detection model to process a sliced image, while $C_{image}$ represents the time required for the classification model to process a CMB region. The dataset used in this section is derived from the CMBs-Private dataset, and comparison results are summarized in Table 10.

Combined with transformer-based classification models, the YOLO series demonstrates superior performance and speed compared to CNN-based architectures. Evaluation of the *FPavg* index indicates that all models have scores exceeding 3, which signifies that the misjudgment rate of CMBs still remains a considerable challenge in this field. However, our proposed framework, as reflected by the $P_{patient}$ index, has attained the highest performance among various combinations. This achievement highlights the effectiveness of our MM-UniCMBs framework in addressing the CMB detection and classification tasks, particularly from a patient-level perspective. Table 11 presents some representative examples of erroneous patient cases. A further analysis of these cases, coupled with clinical diagnostic insights, reveals that the current limitation still lies in the restricted scope of information utilized for identifying challenging cases of CMBs, compared to clinical doctors. Specifically, the absence of detailed patient lifestyle habits and past medical history in the modeling stage forms a significant hurdle in enhancing the accuracy of CMB determinations. This observation underscores the potential values of incorporating a more comprehensive range of patient-specific information into the classification process, suggesting that more holistic input information could improve diagnostic accuracy in the automated detection of CMBs.

**Figure 13:**
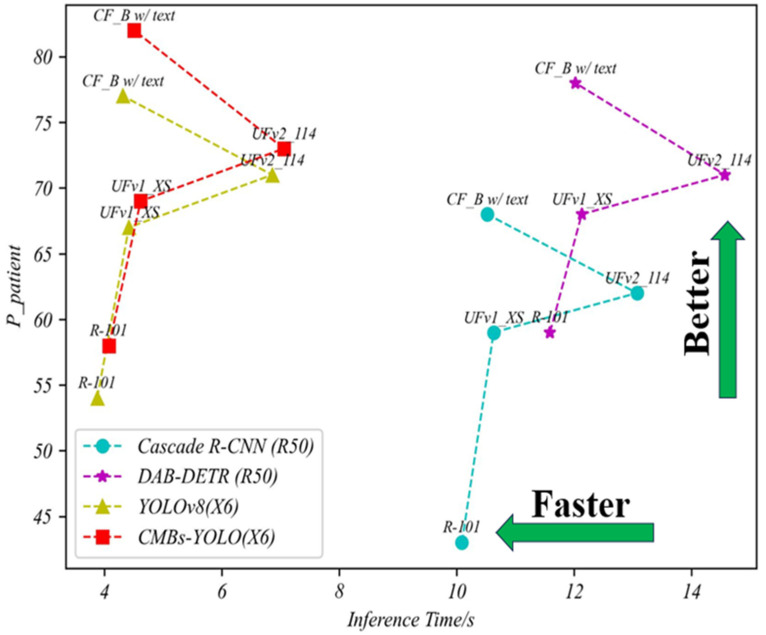
Comparing the trade-off between speed and performance among different models’ combinations in the CMBs-Private testing dataset.

In terms of detection models, our CMBs-YOLO model, coupled with text descriptions input, surpasses the YOLOv8 model in performance. Specifically, it improves upon YOLOv8 by 5% in the $P_{patient}$ metric and by 0.29 in the *FPavg* metric, demonstrating that the CMBs-YOLO effectively enhances CMB classification performance through lowering missing detection rates. Additionally, our CF_B model consistently exhibits lower false detection rates compared to other classification models, regardless of the detection model it is paired with. This improvement is attributed to the integration of patient text description information and the transformer architecture in the CMBs classification process. Moreover, the increased speed of our method can be attributed to its ‘front light and back heavy’ structural design, which allocates more computing resources to the classification task. For readers seeking a more visual and intuitive understanding of these performance metrics, Figure 13 provides a comparative illustration of these aspects.

## 5. Ablation Study

### 5.1. Ablation Study in Detection Stage

In this section, we discuss the ablation experiments conducted during the detection phase using the CMBs-Private dataset. Table 12 illustrates the impact on performance metrics when the CMBs-MHA and NWD components are removed, respectively.

**Figure 14:**
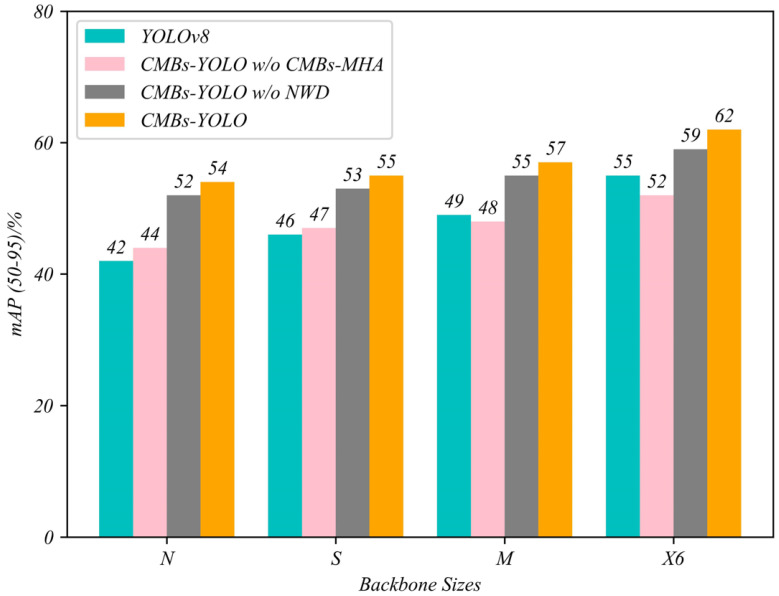
The impact of CMBs-MHA on detection models at various scales in the CMBs-Private testing dataset.

The results in Table 12 clearly demonstrate that the CMBs-MHA module plays a more critical role than the NWD component in our CMB detection model. The removal of the CMBs-MHA module led to a significant decrease in the ${mAP}_{50–95}$ metric, decreasing from 0.62 to 0.44, representing a substantial reduction of 0.18. In contrast, the exclusion of NWD led to a minor decrease of 0.03 in the same metric. This notable disparity underscores the crucial importance of feature modeling capability in the task of detecting CMBs, directly influencing the performance of detection models. Figure 14 visually represents the impact of these changes, providing an intuitive understanding of their significance.

**Figure 15:**
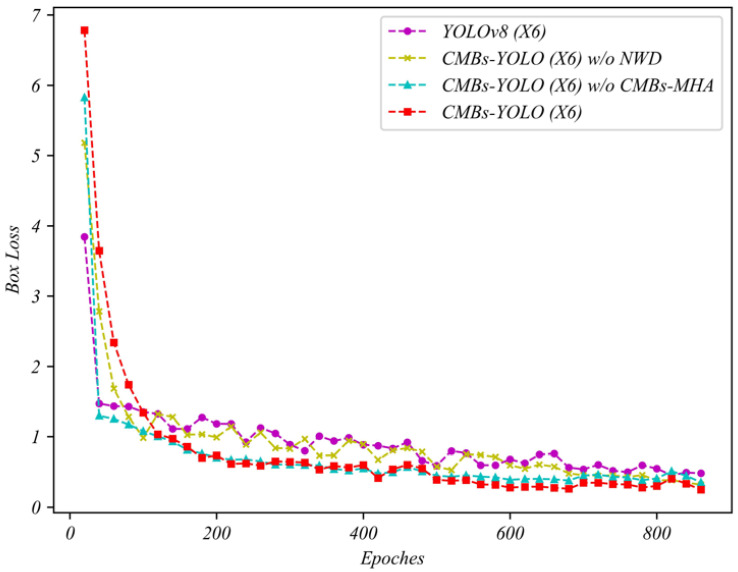
The loss function variation curve during training with the CMBs-Private training dataset.

The incorporation of the NWD loss in detection models is designed to enhance training stability, particularly for small targets. This effect is vividly depicted in Figure 15, where models without NWD loss, such as YOLOv8, exhibit pronounced oscillations in box loss during the training phase. These oscillations lead to increased instability in the network, potentially diminishing the model’s generalization capabilities. In contrast, when NWD is integrated into our CMBs-YOLO model, there is a noticeable improvement in the smoothness and stability of the training process compared to the original configuration. This improvement indicates that the NWD method outperforms IoU-based approaches, especially in dealing with small targets. The increased stability achieved during the training stage is crucial in bolstering the overall robustness and dependability of our detection model, ensuring consistent performance in the CMB detection task.

### 5.2. Ablation Study of Different Input Resolutions in Detection Stage

In Section 4.3.1, we evaluated detection algorithms using fixed 512×512 pixel input resolutions throughout the training and testing phases. In this section, however, we expand the discussion to explore the impact of varying input resolutions on model performance. The dataset used for this analysis is derived entirely from the CMBs-Private dataset.

**Figure 16:**
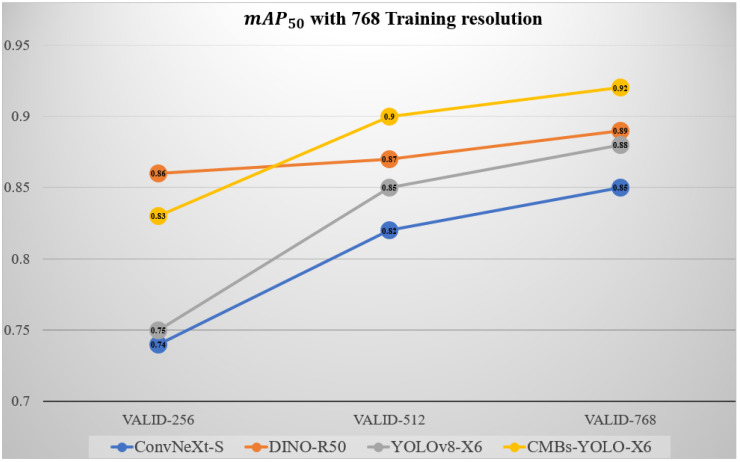
Performance variations of different detection models under the 768-resolution training mode.

Table 13 compares the detection performance across different input resolutions. The results show a positive correlation between increased resolution and improvements in the R metric, while reductions in resolution negatively affect both P and R metrics. When compared with the mAP trends in Figure 16, it is evident that transformer-based models exhibit greater robustness to resolution changes compared to other architectures. Notably, in the 768-resolution training mode, even when the validation resolution was reduced from 768 to 256, the DINO model experienced only a slight decrease of 0.03 in mAP, whereas ConvNeXt showed a larger decline of 0.11.

Although our proposed model is sensitive to resolution changes, it demonstrates improved robustness compared to the original YOLOv8 structure. For instance, in the 768-resolution training mode, our model’s mAP decreased by 0.09, whereas YOLOv8 exhibited a drop of 0.13. This improvement can be attributed to the enhanced feature modeling capability in MRI. The saliency modeling module enhances the network’s ability to differentiate between CMBs and their surrounding background. Additionally, the integration of the NWD loss function during training increases the model’s sensitivity to small targets, further mitigating the impact of resolution changes.

**Figure 17:**
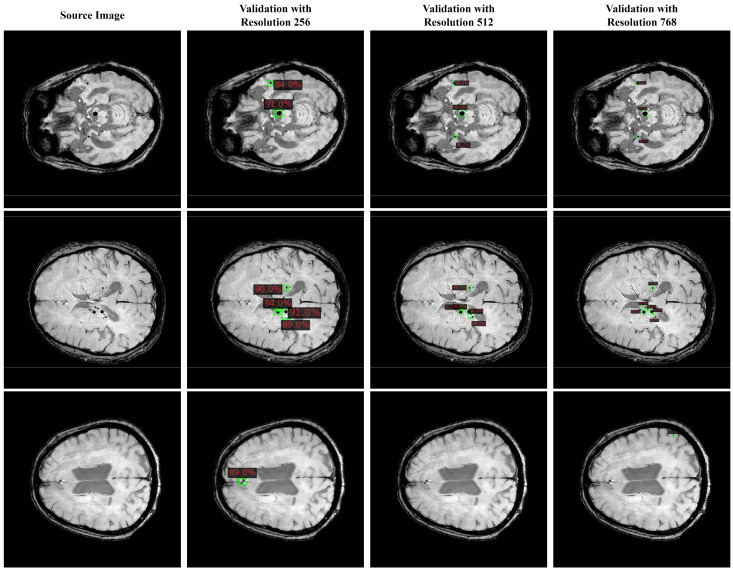
Impact of resolution changes on model prediction scores.

It is important to note that inconsistencies between training and testing resolutions can introduce variability in the model’s detection of identical targets, as shown in Figure 17. This occurs because the models are not exposed to randomly resized augmentations during training. Addressing this limitation through strategies such as multi-scale training or data augmentation could enhance the model’s generalization and robustness across varying resolutions. 

### 5.3. Ablation Study of Different Input Resolutions in Classification Stage

During the detection phase, we obtained numerous candidate boxes for CMBs, each varying in size. Section 4.3.2 presents a performance comparison of classification models in fixed-resolution mode. In this section, we will discuss the impact of input resolution on classification models. To facilitate this analysis, we have designed two comparison modes: (1) fixed-resolution training with fixed-resolution testing and (2) dynamic-resolution training with dynamic-resolution testing. In the fixed-resolution mode, the input 3D images are resized to a standard fixed size, whereas in the dynamic-resolution mode, the input image retains the dimensions of the original detection box. The comparison results for these two modes, utilizing the CMBs-YOLO (X6) model during the detection stage, are summarized in Table 14.

**Figure 18:**
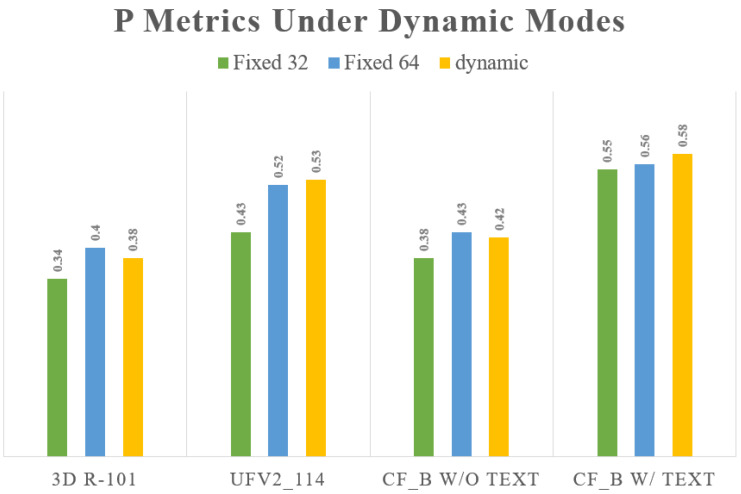
Performance variations of different classification models in dynamic mode.

In fixed-resolution mode, increasing the input resolution can enhance the model’s performance. Conversely, combining high-resolution training with low-resolution prediction leads to a notable decline in the performance of all methods, indicating a strong coupling between training and prediction. In dynamic-resolution mode, the performance of different models exhibits variability, suggesting that this mode is less suitable for the CMB classification task. However, the incorporation of text information in dynamic mode improves the model’s robustness to variations in resolution. For instance, in the dynamic mode of the CF model, CF-B with text enhanced the P index by 0.16 compared to CF-B without text information. This underscores the importance of incorporating additional feature dimensions in CMB classification to enhance accuracy. Figure 18 provides a more intuitive comparison of the performance changes observed across different models in dynamic mode.

### 5.4. Ablation Study in Image Branch

In the initial design of our classification model, a 3D CNN structure was employed in the image branch for feature extraction from 3D CMB regions. This section presents an alternative approach, wherein 2D convolution is used alongside 2D images in the image branch. While this method still incorporates text descriptions and transformer structures, it introduces a variation in the treatment of 3D inputs. Specifically, these 3D images are first averaged to create 2D representations, which are then processed through a 2D convolutional network in the image branch. The dataset used in this section is derived from the CMBs-Private dataset, and the results of this comparative approach are detailed in Table 15.

The results presented in Table 15 clearly demonstrate that the classification performance of the 2D method is generally inferior to that of the 3D approach. Notably, this performance decline is more pronounced in CNN-based methods compared to the CF method. For instance, the 2D-R101 model exhibits a 13% decrease in the P indicator relative to its 3D counterpart (3D-R101), whereas the decrease for the 2D-CF_B model is just 6% when compared to the 3D-CF_B model. This suggests that the integration of textual information effectively mitigates the limitations of relying solely on image data. Additionally, the incorporation of a transformer structure enhances the network’s ability to process feature relationships relevant to CMBs and their mimics. Table 16 presents specific cases that illustrate the tendency of 2D methods to produce erroneous results.

As shown in Table 16, the comparative results clearly highlight the limitations of 2D methods when applied in the image branch, particularly their reduced capacity to extract information across different slice images due to the averaging operations involved. This limitation often leads to the misidentification of false CMBs. For instance, in Case 3, cerebral calcification is clearly visible in the phase image $P_{i}$, but this distinction is obscured in the averaged images, resulting in misclassification. In contrast, 3D methods are more effective at extracting feature information from multiple slice images, which enables them to outperform 2D approaches and deliver more accurate classification results.

### 5.5. Ablation Study of CLIP Model

**Figure 19:**
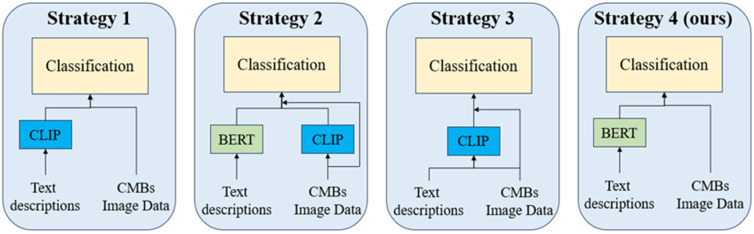
Different classification network structures based on CLIP.

**Figure 20:**
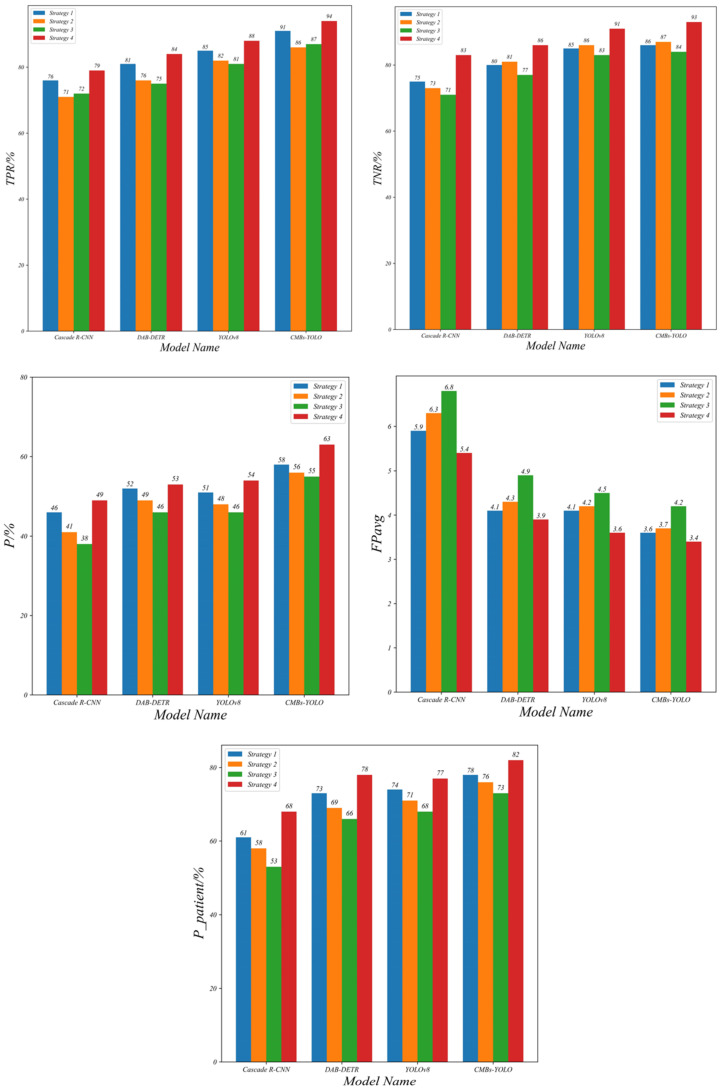
Performance comparison of different strategies under CLIP.

In the initial design of our classification model, we utilized BERT to transform our text prompts into word embedding vectors. This section will discuss the application of the CLIP model [44], examining its effectiveness in classifying CMBs. CLIP, renowned for its proficiency in encoding both text and images, inspired us to devise four different comparison strategies. Specifically, in the text branch of our model, we substitute BERT’s text encoding with CLIP’s encoding mechanism. For the image branch, our approach involves incorporating CLIP’s image encoding features into our pre-existing 3D image branch. These alterations to the model’s structure are illustrated in Figure 19. The dataset used in this section is derived from the CMBs-Private dataset. The results of implementing these strategies are documented in Table 17 and are also visually represented in Figure 20.

Table 17 reveals that the implementation of the CLIP method has negatively impacted the performance of classification networks, especially those based on CNN detection models. Among these tested strategies, the most pronounced decline in performance was observed in the third approach, followed by the second, while the first strategy showed the least impact. This trend suggests that CLIP’s image encoding may not be well suited for the classification of CMBs, as it tends to reduce the effectiveness of the original model.

Several factors contribute to these adverse effects. Firstly, the training dataset for CLIP may not include the MRI types, limiting its ability to model the CMBs’ features and their mimics. Secondly, due to the limitations of the CLIP model in processing 3D images, it became necessary to encode each slice image independently and subsequently concatenate them together, akin to 2D convolution. This manner significantly reduces the spatial information of CMBs’ features, consequently diminishing the network’s performance, as discussed in previous section. Moreover, even after fine-tuning the CLIP model with our CMB dataset and employing it as a new pretraining model under the second strategy, we observed only a slight improvement in performance compared to the conventional version of CLIP. 

From the perspective of the text branch, CLIP did not outperform BERT. As a multimodal pretraining model, CLIP’s text encoding capabilities are less robust compared to specialized text models like BERT. Consequently, CLIP struggles to accurately model text statements, leading to inferior performance when compared to the BERT approach.

In conclusion, although CLIP is renowned for its versatility and large-scale modeling, it demonstrates limitations in specialized medical tasks, such as the classification of CMBs. Our approach, which combines BERT with a transformer structure, has proven to be more effective in this field.

## 6. Discussion

The detection of CMBs is a critical step in diagnosing many neurological diseases; however, it poses significant challenges for clinical neurologists [1,2]. CMBs are small, unpredictably located, and often surrounded by structures with similar characteristics, making detection particularly challenging for novice neurologists [69]. Even experienced clinicians find manual detection time-consuming and prone to error. The integration of deep learning techniques into the diagnosis of CMBs has emerged as an important interdisciplinary approach, bridging the fields of medicine and computer science [9,37,39,40].

However, existing research has predominantly focused on the use of image data for the detection and classification of CMBs, while overlooking the role of patient-specific textual information on model results [9,11,14]. These approaches typically employ 3D convolution to extract feature information about cerebral hemorrhage points across different slices, followed by detection and classification. This modeling approach diverges significantly from the actual diagnostic processes employed by clinical practitioners, making it challenging for AI models to overcome existing performance limitations in the detection of CMBs. By researching the actual diagnostic workflow of clinical doctors, we have identified several key processes in CMB detection tasks: 1. “Looking”: the clinician first locates potential bleeding areas based on the patient’s CT scans. 2. “Checking”: physicians must integrate information from multiple slice images to differentiate between bleeding and brain calcification. 3. “Asking”: the final judgment is made considering the patient’s specific circumstances, including age, gender, medical history, and lifestyle habits. Based on this understanding, we propose a novel CMB detection framework designed to simulate the actual diagnostic process of clinical practitioners.

**Figure A1:**
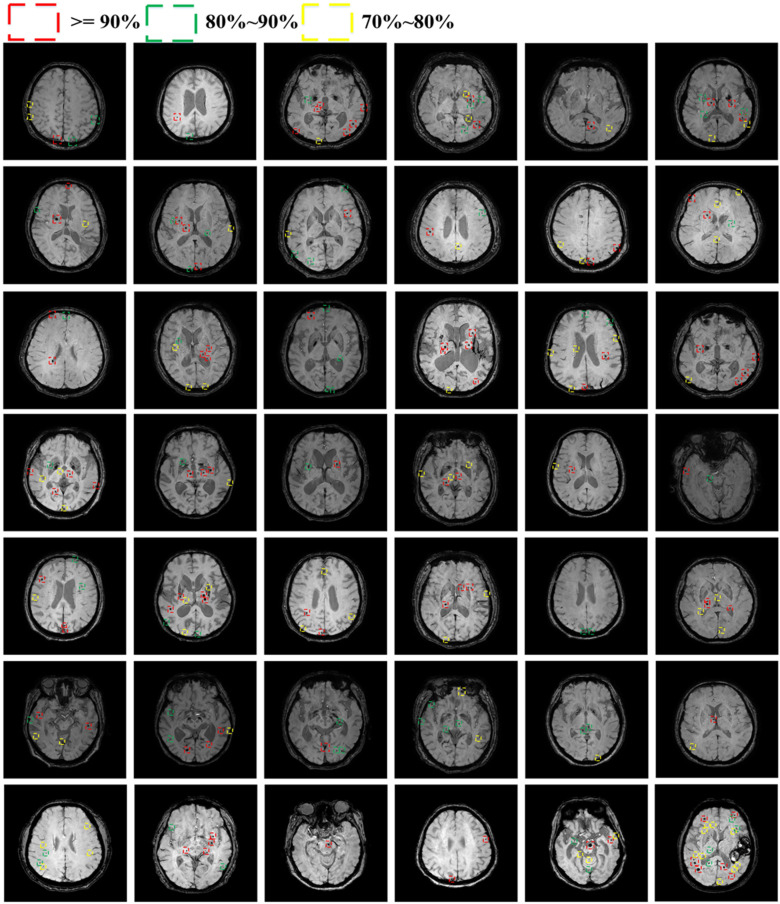
The detection results of CMB-YOLO (X6).

The overall results of this study demonstrate that our proposed framework significantly enhances accuracy compared to existing methods. In the detection stage, as illustrated in Figure 10, the introduction of CMBs-MHA into the detection model enables CMBs-YOLO to strengthen its ability to model the salient features of CMBs. Additionally, Table 6 reveals that our detection approach exhibits strong generalization capabilities, as evidenced by comparisons with various detection models on the CMBs-Public [46] datasets. For classification tasks, we have designed a multimodal network structure that simultaneously integrates image and text data relevant to CMBs. By combining the insights from Table 10 and Figure A1, we conclude that the inclusion of multidimensional features is essential for improving the accuracy of CMBs. Furthermore, a comparison of Table 8 and Table 10 indicates that the multimodal structure achieves superior performance at the patient level, highlighting the advantages of multimodal technology [70,71,72,73] over single-modal model structures [74,75].

Through a series of ablation studies, we validated the hyperparameter optimizations and prediction stability of our framework in the field of CMB detection. Firstly, in the detection phase, we compared the effects of the CMBs-MHA module and the NWD [76,77] module on performance during the training and testing phases. The loss function variations illustrated in Figure 15 indicate that the proposed detection model exhibits strong robustness against underfitting, early convergence, and loss optimization traps. Additionally, we explored the influence of input resolution hyperparameters in the detection networks to further elucidate how different training parameters affect model performance. In the classification network, we first demonstrated the changes in classification performance across various 3D resolutions. As shown in Table 14 and Figure 18, existing classification model theories impose high demands for consistency between training and testing conditions; inconsistent parameter changes can increase the uncertainty of model predictions and consequently reduce performance. We then focused on the impact of text features on CMB classification results. Table 11 presents the role of text information in error cases, highlighting the significance of the text modeling approach in enhancing the accuracy of CMB diagnoses. Building on this, we compared the performance of the CLIP and BERT methods from the perspective of image and text dimensions, as detailed in Table 16 and Figure 19, which underscore the rationality and effectiveness of the methods employed in our framework.

However, our MM-UniCMBs framework exhibits limitations due to its two-stage pipeline design. One concern is that separating the detection and classification stages may disrupt the inherent connection between these tasks, potentially hindering the release of model capabilities. Diwan et al. [78] observed that two-stage detection networks are increasingly being replaced by single-stage structures due to their superior generalization and robustness. Optimization techniques such as Fast Fourier Transform and uncertainty quantification (UQ) [79,80,81] have been explored to accelerate the convergence of object detectors. Abbaszadeh et al. [82] emphasized the importance of UQ methods in assessing model certainty for practical applications and proposed a novel state-of-the-art UQ approach, the Automated Random Deactivating Connective Weights (ARDCWs) ensemble method, for performance validation. In future research, we will investigate the utility of UQ methods in medical recognition tasks to improve the reliability of our models. Another concern is that the decoupled structure of our framework increases training complexity and poses challenges for the automated implementation of CMB detection. In future work, we aim to address these issues by exploring an end-to-end CMB detection method, guided by the principles of multimodal design. Additionally, as more advanced prediction methods are developed, we will investigate the application of technologies such as block combined network structures [83], hybrid block modular models [84], and 3D filtering and block matching [85,86,87] in our medical prediction tasks.

## 7. Conclusions

In this paper, we present a novel multimodal unified framework, termed MM-UniCMBs, specifically designed for the detection of CMBs using MRI and language data. The MM-UniCMBs framework employs a ‘front-light detection stage’ (CMBs-YOLO) and a ‘back-heavy classification stage’ (CMBsFormer). To enhance the representation of weak features, we propose a multi-head attention module, CMBs-MHA, specifically designed to capture salient features that differentiate CMBs from surrounding tissues. To improve the robustness of box regression for small targets, we introduce the NWD loss function, which enhances stability compared to traditional IoU-based methods. Comparative analysis shows that CMBs-YOLO achieves superior detection performance over existing approaches. In the classification phase, we introduce CMBsFormer, a multimodal classification network designed to address the limitations of previous studies that solely relied on single-image data. CMBsFormer integrates multidimensional patient descriptors during the classification process, combining both visual data and patient-specific information. This framework merges the ‘looking’ step, which analyzes MRI images, with the ‘asking’ step, incorporating patient features such as age and gender. This integration enriches the model’s feature space and enhances interpretability, helping to reduce the disparity between AI-driven methods and clinical diagnostic procedures. Additionally, the incorporation of a transformer structure further strengthens the network’s ability to synthesize information across image and text modalities, providing superior adaptability compared to conventional CNN architectures. CF is flexible and scalable, capable of processing both single-modality image inputs and multimodal image–text inputs. Implemented in the diagnostic process, this framework achieved 82% accuracy and a diagnostic speed of 5 s. Moreover, this framework serves as a valuable tool for less experienced clinicians, offering an auxiliary reference for CMBs diagnosis.

## Figures and Tables

**Table 1:** The distribution of CMBs-Public dataset.

CMBs-Public	Number of Patients	Number of Patients with CMBs	Average Age of Patients with CMBs	Average Age of Patients without CMBs	Number of Slices per Patient	Slice Image Resolution	Number of CMBs Bounding Boxes	Male Proportion	Female Proportion
Training set	50	37	×	×	35	512 × 512	353	×	×
Validation set	7	4	×	×	35	512 × 512	43	×	×
Testing set	15	9	×	×	35	512 × 512	102	×	×

**Table 2:** The distribution of CMBs-Private dataset.

CMBs-Private	Number of Patients	Number of Patients with CMBs	Average Age of Patients with CMBs	Average Age of Patients without CMBs	Number of Slices per Patient	Slice Image Resolution	Number of CMBs Bounding Boxes	Male Proportion	Female Proportion
Training set	400	178	66	57	130	512 × 512	8015	61%	39%
Validation set	50	17	68	61	130	512 × 512	687	54%	46%
Testing set	150	56	64	59	130	512 × 512	2432	66%	34%

**Table 3:** The comparison of different loss functions.

Loss Name	Value (A, B)	Value (A, C)	Trend	Value (D, E)	Value (D, F)	Trend
IoU [12]	0.29	0.06	−0.23	0.92	0.85	−0.07
GIoU [51]	0.16	−0.26	−0.42	0.92	0.85	−0.07
DIoU [12]	0.29	0.06	−0.23	0.92	0.85	−0.07
CIoU [12]	0.29	0.06	−0.23	0.92	0.85	−0.07
NWD	0.9	0.8	−0.1	0.9	0.8	−0.1

**Table 4:** The format of text prompts and their corresponding descriptions.

Text Prompts	Descriptions
This is a {*male/female*} patient with {} years old.	Describe the patient’s gender and age.
Target length and width are {} and {} pixels.	Describe the length and width of the detected CMBs.
The detection status of the {*previous/next*} frame at the same position is {*yes/no*}.	Describe the detection status of CMBs in the previous and next slice images at the same position.

**Table 5:** The training configuration of detection models.

Method Name	Model Name	Proposed Year	Backbone	Dropout Rate	Batch Size	Learning Rate	Epoches
**CNN**	Faster R-CNN [47]	2015	R-50 [55]	0.0	8	0.001	30
	RetinaNet [56]	2017	R-50	0.0	8	0.001	30
	Cascade R-CNN [57]	2018	R-50	0.0	4	0.001	45
	CenterNet [58]	2019	R-50	0.0	8	0.001	30
	Dynamic R-CNN [59]	2020	R-50	0.0	8	0.001	30
	Sparse R-CNN [60]	2021	R-50	0.0	8	0.001	30
	ConvNeXt [61]	2022	T	0.5	8	0.001	30
			S	0.5	8	0.001	30
**Transformer**	DETR [48]	2020	R-50	0.0	4	0.0001	50
	Deformable DETR [62]	2021	R-50	0.0	4	0.0001	50
	DAB-DETR [63]	2022	R-50	0.0	4	0.0001	50
	DINO [64]	2023	R-50	0.0	4	0.0001	50
**YOLO**	YOLOv3 [65]	2018	DarkNet-53	0.0	8	0.001	30
	YOLOX [66]	2021	S	0.0	8	0.001	30
	YOLOv8	2023	N	0.3	32	0.001	30
			S	0.3	32	0.001	30
			M	0.2	16	0.001	30
			X6	0.2	8	0.001	30
	CMBs-YOLO		N	0.3	32	0.001	30
			S	0.3	32	0.001	30
			M	0.2	16	0.001	30
			X6	0.2	8	0.001	30

**Table 6:** The performance comparison in the CMBs-Private testing dataset.

Method Name	Model Name	Backbone	Parameters (MB)	P	R	${mAP}_{50}$	${mAP}_{50–95}$	Inference Time/ms
**CNN**	Faster R-CNN	R-50	41.75	0.47	0.38	0.43	0.32	52.96
	RetinaNet	R-50	37.96	0.49	0.43	0.49	0.36	47.34
	Cascade R-CNN	R-50	69.39	0.56	0.49	0.51	0.42	60.83
	CenterNet	R-50	32.29	0.47	0.53	0.55	0.36	25.32
	Dynamic R-CNN	R-50	41.75	0.68	0.57	0.61	0.38	34.17
	Sparse R-CNN	R-50	106.29	0.75	0.63	0.68	0.41	57.42
	ConvNeXt	T	48.09	0.79	0.66	0.73	0.43	32.95
		S	106.35	0.88	0.75	0.81	0.52	65.13
**Transformer**	DETR	R-50	41.58	0.84	0.77	0.84	0.49	54.67
	Deformable DETR	R-50	40.12	0.86	0.81	0.86	0.52	65.36
	DAB-DETR	R-50	44.27	0.89	0.83	0.87	0.54	72.39
	DINO	R-50	47.55	0.92	0.91	0.88	0.64	47.69
**YOLO**	YOLOv3	DarkNet-53	61.95	0.57	0.51	0.48	0.39	32.54
	YOLOX	S	8.97	0.81	0.72	0.75	0.43	14.61
	YOLOv8	N	2.87	0.72	0.63	0.66	0.42	6.2
		S	10.62	0.79	0.68	0.74	0.46	7.3
		M	24.66	0.88	0.71	0.77	0.49	8.9
		X6	92.81	0.91	0.78	0.86	0.55	13.1
	CMBs-YOLO	N	3.33	0.78	0.69	0.75	0.51	7.6
		S	12.44	0.87	0.79	0.83	0.55	8.4
		M	27.53	0.91	0.82	0.87	0.57	11.3
		X6	95.43	0.94	0.86	0.91	0.62	14.6

**Table 7:** The performance comparison in the CMBs-Public testing dataset.

Method Name	Model Name	Backbone	P	R	${mAP}_{50}$	${mAP}_{50–95}$
**CNN**	Faster R-CNN	R-50	0.41	0.22	0.35	0.26
	RetinaNet	R-50	0.54	0.29	0.47	0.31
	Cascade R-CNN	R-50	0.58	0.51	0.46	0.32
	CenterNet	R-50	0.64	0.43	0.57	0.34
	Dynamic R-CNN	R-50	0.68	0.57	0.61	0.43
	Sparse R-CNN	R-50	0.68	0.39	0.65	0.52
	ConvNeXt	T	0.77	0.47	0.65	0.53
		S	0.82	0.53	0.78	0.59
**Transformer**	DETR	R-50	0.64	0.41	0.57	0.52
	Deformable DETR	R-50	0.66	0.39	0.63	0.61
	DAB-DETR	R-50	0.68	0.43	0.64	0.61
	DINO	R-50	0.81	0.48	0.77	0.65
**YOLO**	YOLOv3	DarkNet-53	0.43	0.31	0.38	0.32
	YOLOX	S	0.72	0.49	0.63	0.54
	YOLOv8	N	0.61	0.37	0.58	0.49
		S	0.68	0.48	0.64	0.52
		M	0.75	0.52	0.71	0.57
		X6	0.79	0.55	0.74	0.63
	CMBs-YOLO	N	0.66	0.43	0.61	0.55
		S	0.71	0.51	0.68	0.59
		M	0.78	0.57	0.73	0.61
		X6	0.83	0.62	0.79	0.68

**Table 8:** The performance comparison of classification models.

Method Name	Model Name	TPR	TNR	P	Parameter (MB)	Inference Time/ms
**3D-CNN**	R-18	0.64	0.69	0.14	31.6	7.8
	R-50	0.72	0.75	0.23	60.5	10.1
	R-101	0.85	0.87	0.36	117.9	21.8
**Transformer**	UFv1_XXS	0.83	0.84	0.46	10.1	27.2
	UFv1_XS	0.84	0.85	0.47	16.3	33.1
	UFv2_B16	0.88	0.86	0.54	108.5	36.7
	UFv2_114	0.89	0.88	0.56	336.9	51.6
**CF**	CF_S w/o text	0.86	0.85	0.49	26.68	12.7
	CF_B w/o text	0.89	0.86	0.51	107.15	18.86
	CF_S w/text	0.92	0.91	0.59	30.1	18.6
	CF_B w/text	0.94	0.93	0.63	114.5	26.12

**Table 9:** Visual comparison of slice input images and texts in classification.

	Input Images	Gender	Age	Ground Truth	R101	UFv1_XS	UFv2_114	CF_B w/o text	CF_B w/text
Patient 1	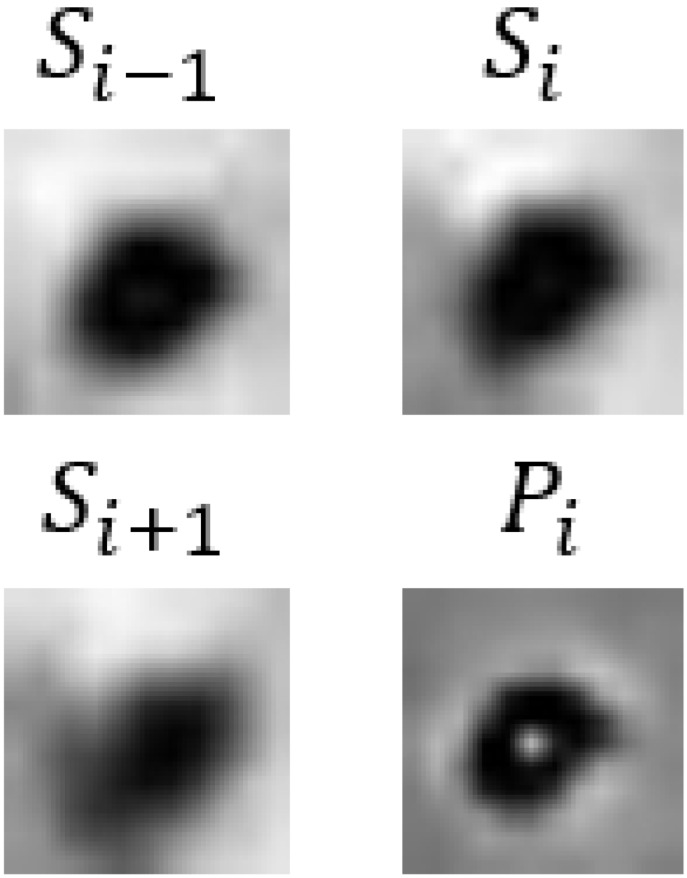	Female	55	Not CMBs	CMBs	CMBs	CMBs	CMBs	Not CMBs
Patient 2	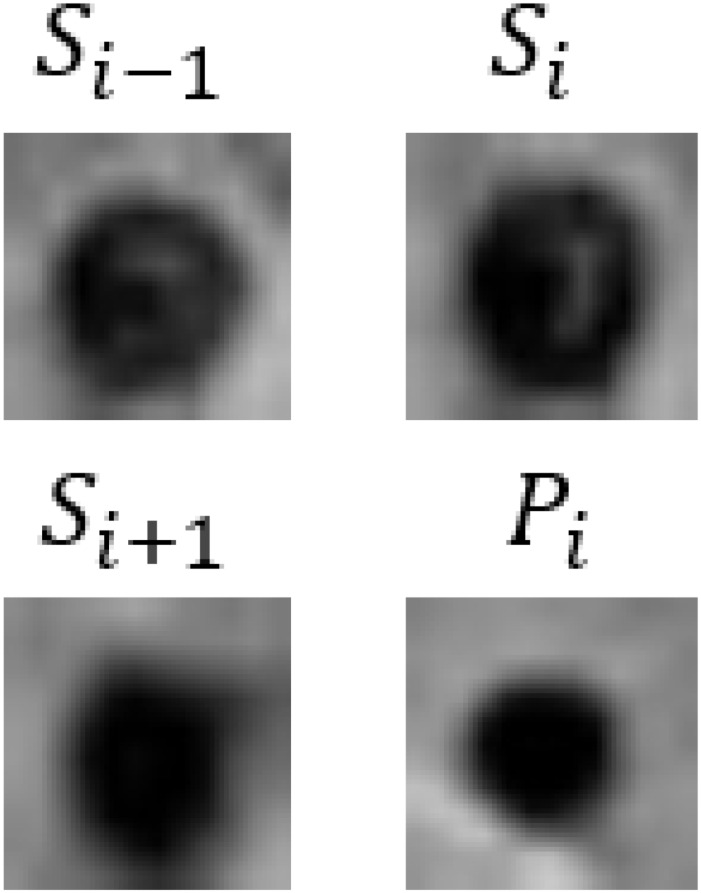	Male	68	CMBs	CMBs	CMBs	CMBs	CMBs	CMBs
Patient 3	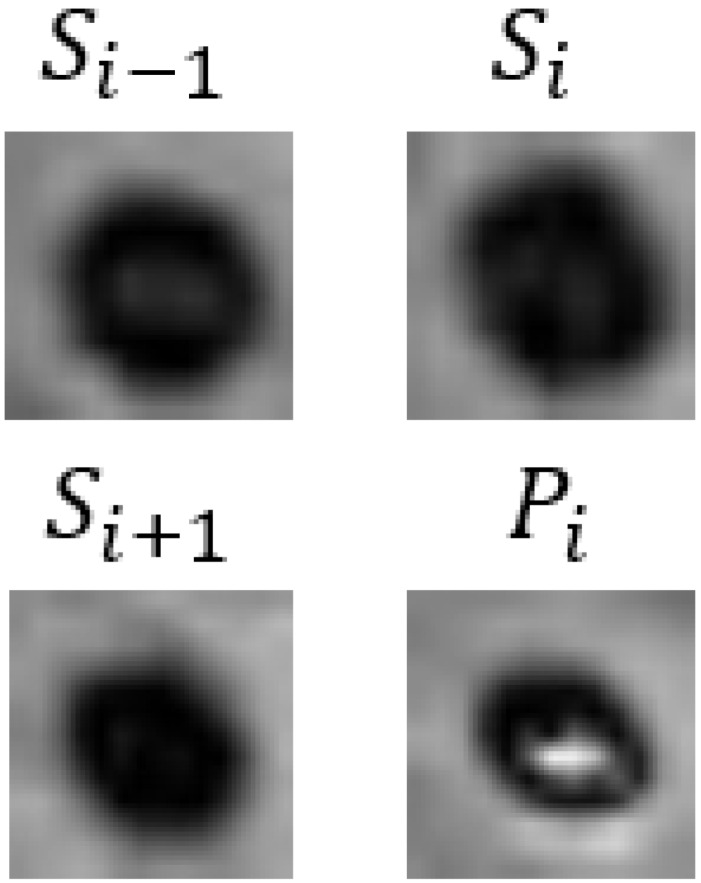	Female	57	Not CMBs	CMBs	CMBs	CMBs	CMBs	Not CMBs

**Table 10:** The comparison of different models’ combinations.

Detection Model	Classification Model	FPavg	$P_{patient}$	Total Parameters/MB	Total Inference Time/s
**Cascade R-CNN (R50)**	R-101	10.87	0.43	187.29	10.09
	UFv1_XS	7.98	0.59	85.69	10.63
	UFv2_114	7.06	0.62	406.29	13.07
	CF_B w/text	5.49	0.68	183.86	10.52
**DAB-DETR** **(R50)**	R-101	9.63	0.51	162.17	11.59
	UFv1_XS	7.14	0.68	60.57	12.13
	UFv2_114	5.67	0.69	381.17	14.57
	CF_B w/text	3.98	0.78	158.77	12.02
**YOLOv8** **(X6)**	R-101	8.19	0.54	210.71	3.88
	UFv1_XS	6.33	0.67	109.11	4.42
	UFv2_114	5.72	0.71	429.71	6.86
	CF_B w/text	3.66	0.77	207.31	4.32
**CMBs-YOLO** **(X6)**	R-101	8.42	0.58	213.03	4.08
	UFv1_XS	6.97	0.69	111.73	4.62
	UFv2_114	6.21	0.73	432.33	7.06
	CF_B w/text	3.37	0.82	209.93	4.51

**Table 11:** Instances of incorrect judgments in CMB classification stage.

	Image	Enlarged View	Age	Gender	Lifestyle Habits	Medical History	Clinical Diagnostic Results	Our Results
**Patient 1**	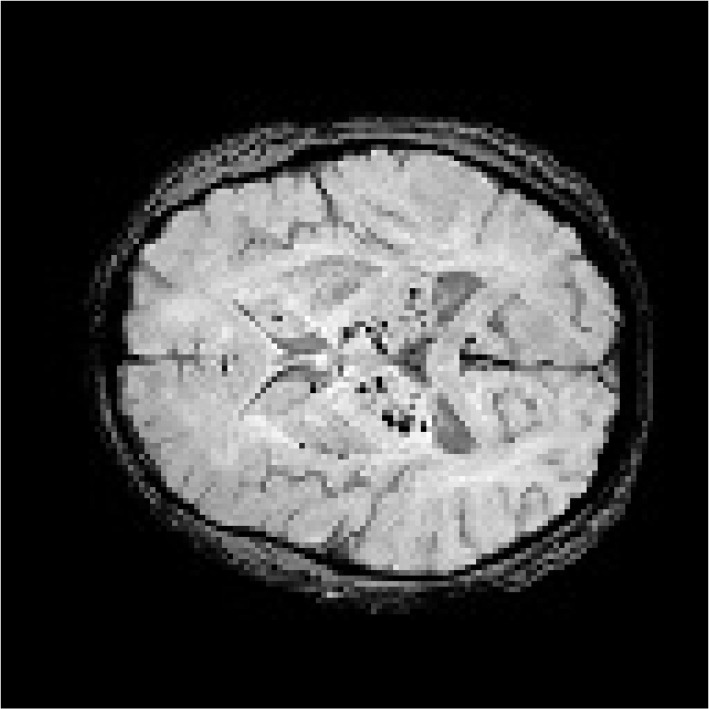	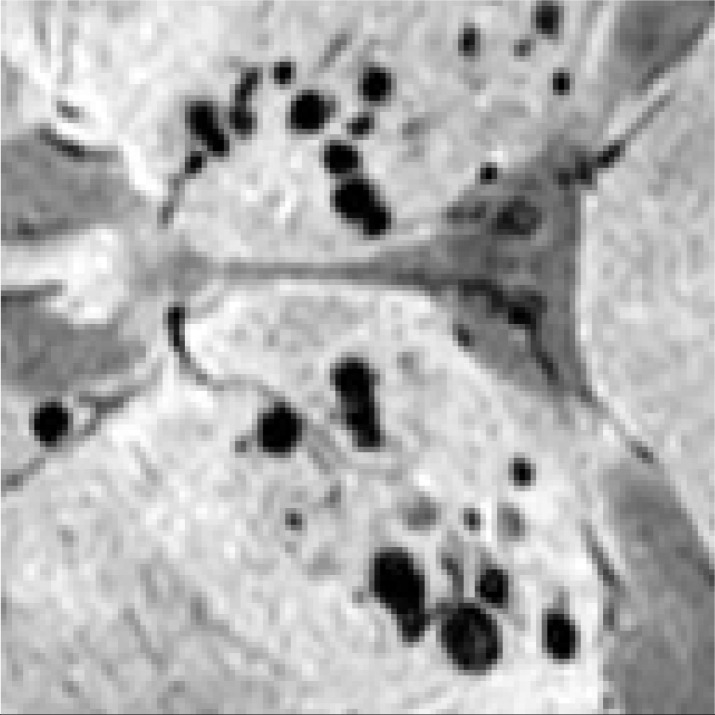	53	Female	×	CADASIL	CMBs	Not CMBs
**Patient 2**	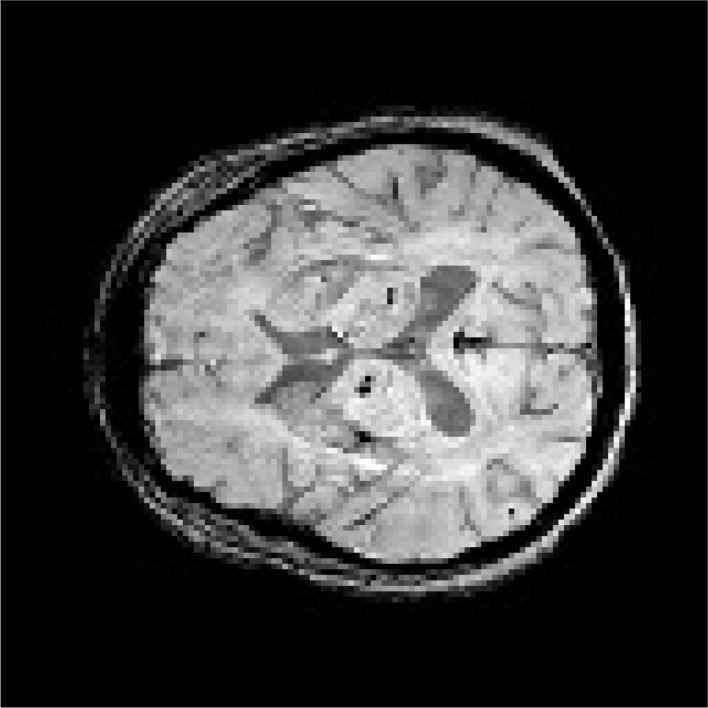	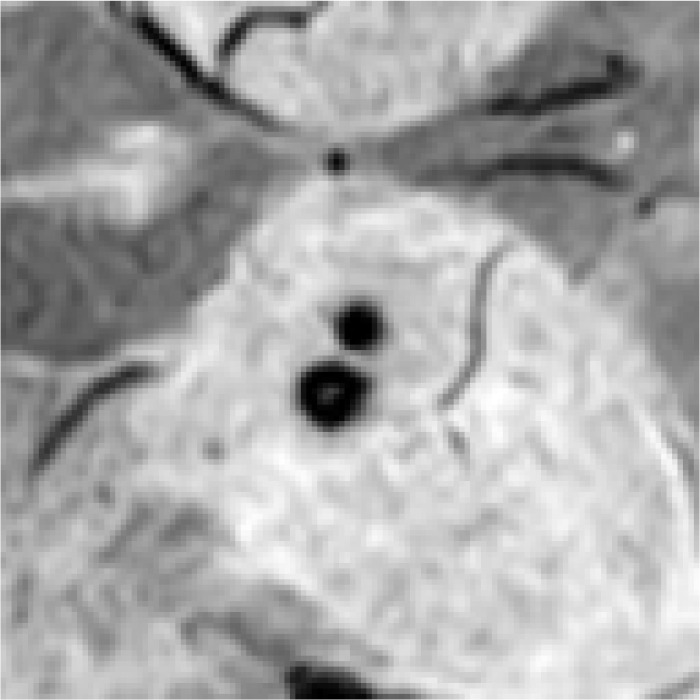	55	Male	Drinking and Smoking	Hypertension	CMBs	Not CMBs
**Patient 3**	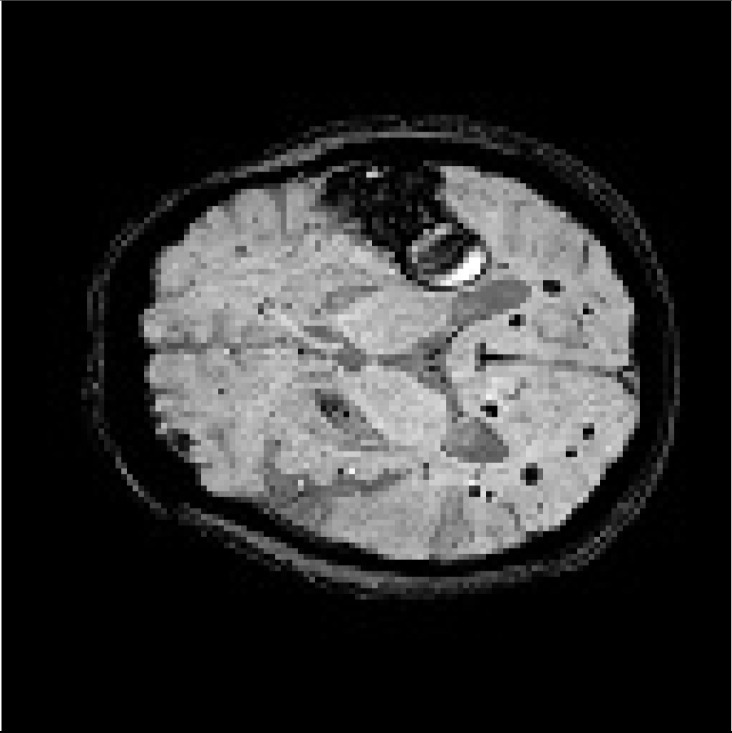	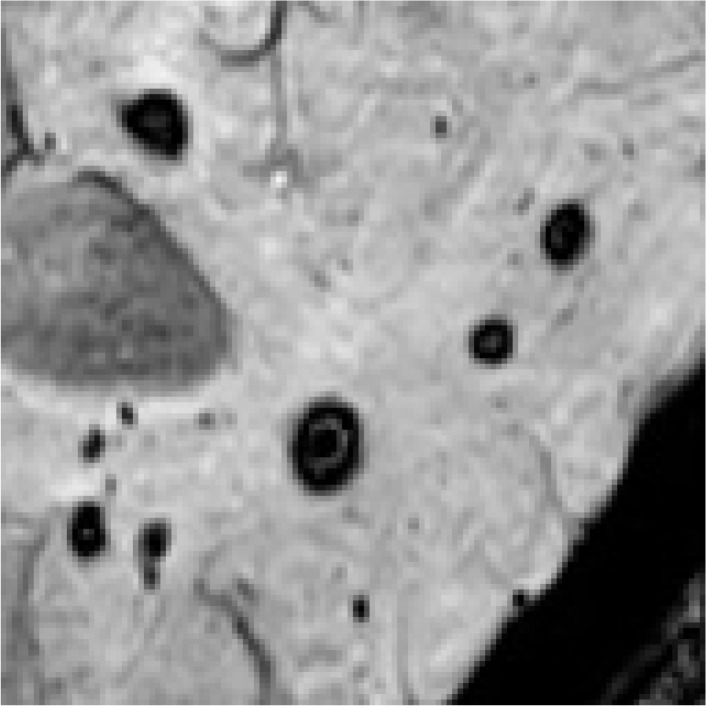	78	Male	×	×	Cerebral Calcification (Not CMBs)	CMBs

**Table 12:** The impact of CMBs-MHA and NWD on the detection model.

Model Name	Backbone	Box Loss	P	R	${mAP}_{50}$	${mAP}_{50–95}$
**YOLOv8**	N	0.49	0.72	0.63	0.66	0.42
	S	0.47	0.79	0.68	0.74	0.46
	M	0.43	0.88	0.71	0.77	0.49
	X6	0.40	0.91	0.78	0.86	0.55
**CMBs-YOLO** **w/o** **NWD**	N	0.43	0.76	0.68	0.72	0.52
	S	0.41	0.86	0.78	0.81	0.53
	M	0.36	0.90	0.79	0.85	0.55
	X6	0.36	0.92	0.87	0.89	0.59
**CMBs-YOLO** **w/o** **CMBs-MHA**	N	0.43	0.73	0.64	0.67	0.44
	S	0.42	0.81	0.67	0.75	0.47
	M	0.37	0.86	0.73	0.78	0.48
	X6	0.33	0.88	0.79	0.85	0.52
**CMBs-YOLO**	N	0.39	0.78	0.69	0.75	0.54
	S	0.37	0.87	0.79	0.83	0.55
	M	0.32	0.91	0.82	0.87	0.57
	X6	0.31	0.94	0.86	0.91	0.62

**Table 13:** The performance comparison of input resolutions.

Model Name	Backbone	Train Resolution	Validation Resolution	P	R	${mAP}_{50}$	${mAP}_{50–95}$
ConvNeXt	S	512×512	256×256	0.80	0.66	0.76	0.49
			512×512	0.83	0.75	0.81	0.52
			768×768	0.88	0.77	0.82	0.54
		768×768	256×256	0.82	0.57	0.74	0.43
			512×512	0.86	0.74	0.82	0.52
			768×768	0.89	0.81	0.85	0.57
DINO	R50	512×512	256×256	0.91	0.85	0.86	0.61
			512×512	0.92	0.89	0.88	0.64
			768×768	0.92	0.88	0.87	0.64
		768×768	256×256	0.89	0.88	0.86	0.63
			512×512	0.92	0.91	0.87	0.64
			768×768	0.93	0.91	0.89	0.66
YOLOv8	X6	512×512	256×256	0.83	0.74	0.76	0.52
			512×512	0.91	0.78	0.86	0.55
			768×768	0.92	0.78	0.85	0.54
		768×768	256×256	0.81	0.72	0.75	0.49
			512×512	0.88	0.79	0.85	0.56
			768×768	0.92	0.81	0.88	0.57
CMBs-YOLO	X6	512×512	256×256	0.88	0.81	0.85	0.57
			512×512	0.93	0.86	0.91	0.62
			768×768	0.94	0.87	0.90	0.61
		768×768	256×256	0.86	0.83	0.83	0.55
			512×512	0.93	0.88	0.90	0.62
			768×768	0.95	0.89	0.92	0.62

**Table 14:** The comparison results of 2D images input in classification.

Method Name	Model Name	Training Mode/Resolution	Testing Mode/Resolution	TPR	TNR	P
**3D-CNN**	R-101	Fixed 32×32×32	Fixed 32×32×32	0.69	0.71	0.31
			Fixed 64×64×64	0.83	0.84	0.34
			Dynamic	0.82	0.86	0.33
		Fixed 64×64×64	Fixed 32×32×32	0.79	0.82	0.32
			Fixed 64×64×64	0.85	0.87	0.36
			Dynamic	0.84	0.83	0.35
		Dynamic	Fixed 32×32×32	0.72	0.69	0.34
			Fixed 64×64×64	0.78	0.76	0.40
			Dynamic	0.76	0.74	0.38
**Transformer**	UFv2_114	Fixed 32×32×32	Fixed 32×32×32	0.77	0.79	0.39
			Fixed 64×64×64	0.81	0.83	0.42
			Dynamic	0.80	0.84	0.41
		Fixed 64×64×64	Fixed 32×32×32	0.84	0.83	0.49
			Fixed 64×64×64	0.89	0.88	0.56
			Dynamic	0.85	0.81	0.51
		Dynamic	Fixed 32×32×32	0.78	0.79	0.43
			Fixed 64×64×64	0.86	0.83	0.52
			Dynamic	0.85	0.86	0.53
**CF**	CF_B w/o text	Fixed 32×32×32	Fixed 32×32×32	0.82	0.81	0.41
			Fixed 64×64×64	0.83	0.82	0.41
			Dynamic	0.81	0.80	0.39
		Fixed 64×64×64	Fixed 32×32×32	0.83	0.82	0.45
			Fixed 64×64×64	0.89	0.86	0.51
			Dynamic	0.84	0.81	0.41
		Dynamic	Fixed 32×32×32	0.79	0.75	0.38
			Fixed 64×64×64	0.83	0.79	0.43
			Dynamic	0.82	0.80	0.42
	CF_B w/text	Fixed 32×32×32	Fixed 32×32×32	0.88	0.87	0.56
			Fixed 64×64×64	0.89	0.88	0.58
			Dynamic	0.85	0.87	0.55
		Fixed 64×64×64	Fixed 32×32×32	0.90	0.89	0.57
			Fixed 64×64×64	0.94	0.93	0.63
			Dynamic	0.92	0.91	0.59
		Dynamic	Fixed 32×32×32	0.87	0.85	0.55
			Fixed 64×64×64	0.91	0.88	0.56
			Dynamic	0.91	0.87	0.58

**Table 15:** The comparison results of 2D images input in classification.

Method Name	Classification Model Name	TPR	TNR	P
**2D-CNN**	R-18	0.52	0.49	0.07
	R-50	0.61	0.62	0.15
	R-101	0.73	0.76	0.23
**3D-CNN**	R-18	0.64	0.69	0.14
	R-50	0.72	0.75	0.23
	R-101	0.85	0.87	0.36
**2D-CF**	CF_S w/text	0.87	0.84	0.55
	CF_B w/text	0.89	0.87	0.57
**3D-CF**	CF_S w/text	0.92	0.91	0.59
	CF_B w/text	0.94	0.93	0.63

**Table 16:** The difference between 2D and 3D images.

	2D Input Image (Averaging)	3D Input Images	Ground Truth	2D-R101	3D-R101	2D-CF_B	3D-CF_B
**Case 1**	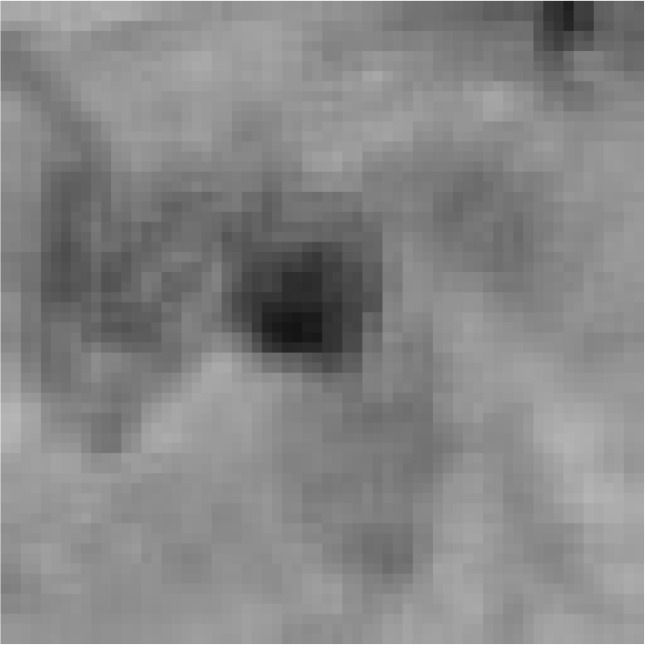	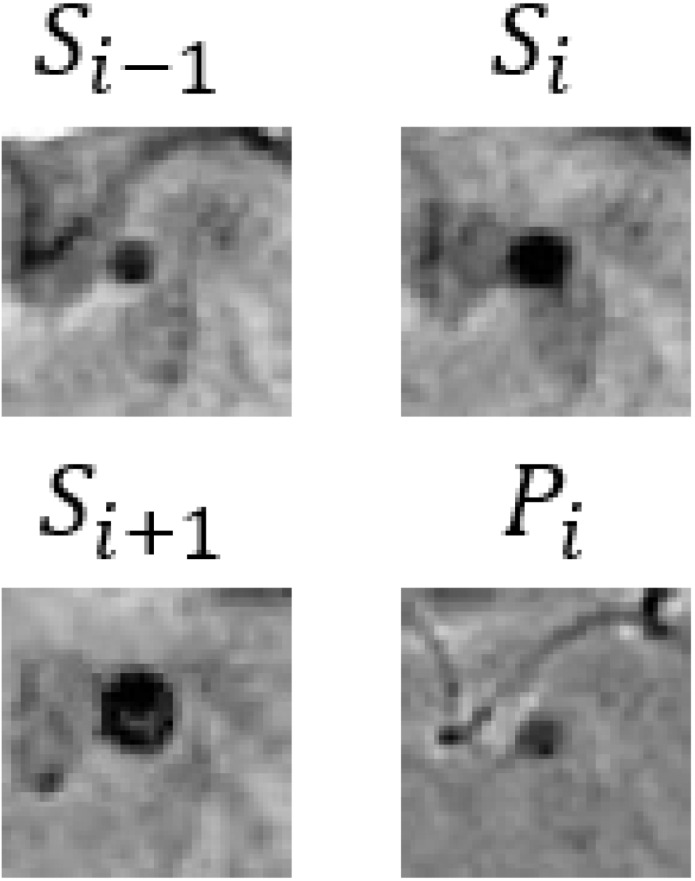	CMBs	Not CMBs	CMBs	CMBs	CMBs
**Case 2**	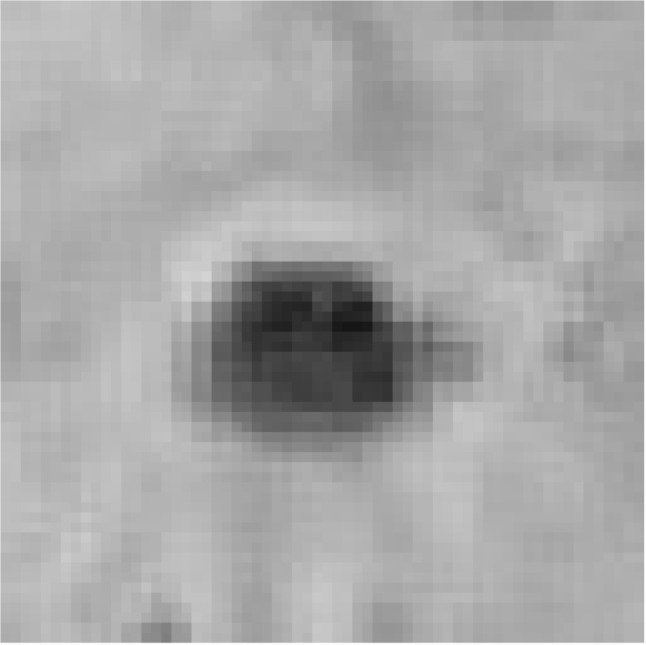	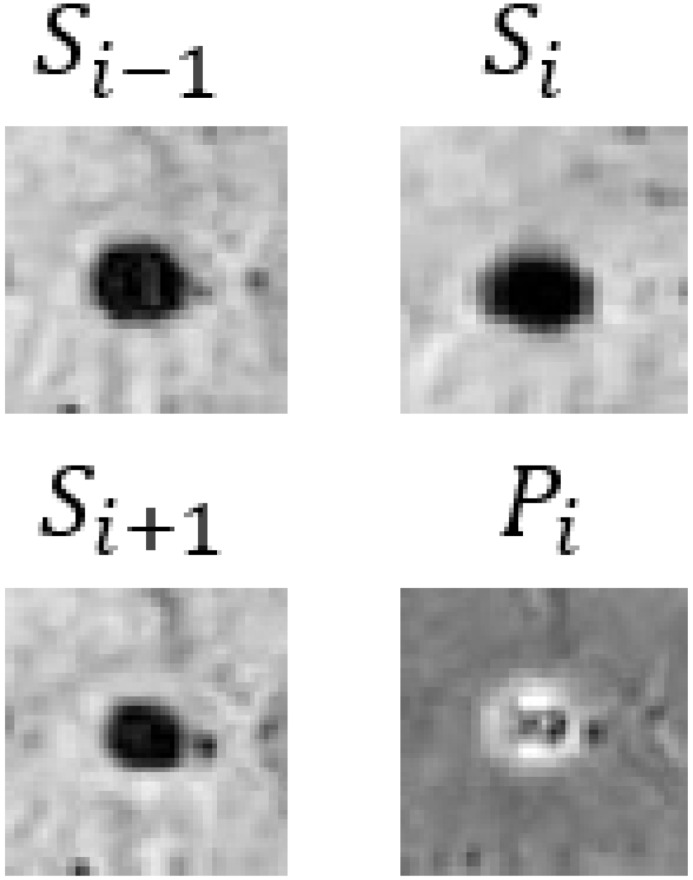	Cerebral Calcification (Not CMBs)	CMBs	Not CMBs	CMBs	Not CMBs
**Case 3**	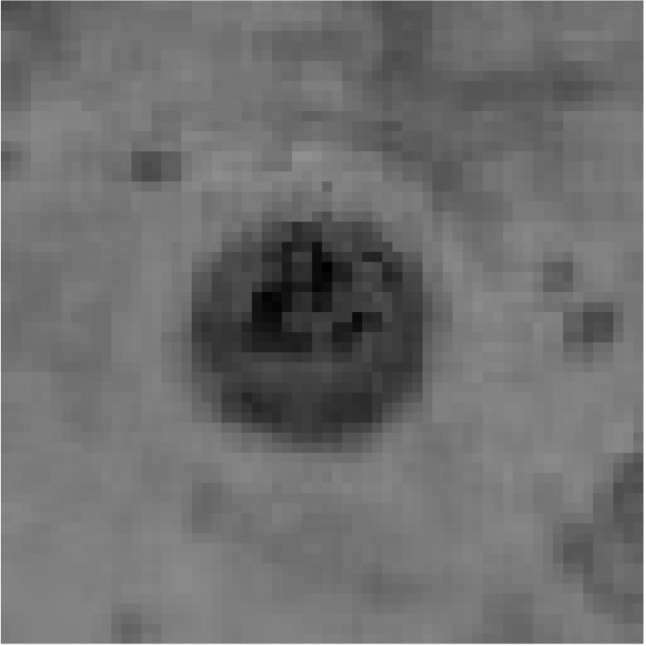	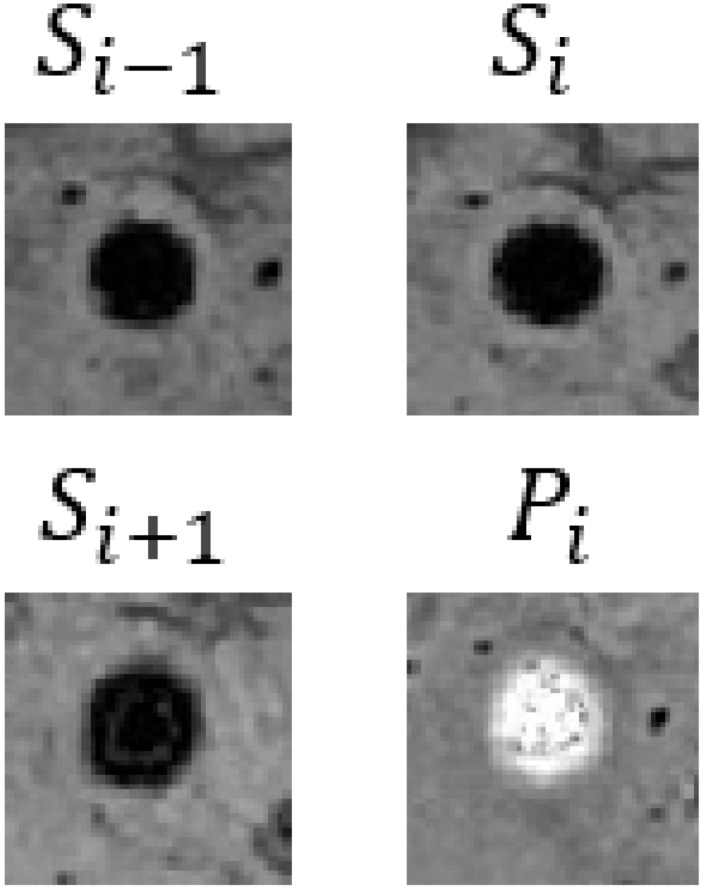	Cerebral Calcification (Not CMBs)	CMBs	Not CMBs	CMBs	Not CMBs

**Table 17:** The comparison of different strategies in the CMBs-Private testing dataset.

Detection Model	Classification Model	Comparison Strategy	TPR	TNR	P	FPavg	$P_{patient}$
**Cascade** **R-CNN (R50)**	CF_B	1	0.76	0.75	0.46	5.92	0.61
		2	0.71	0.73	0.41	6.31	0.58
		3	0.72	0.71	0.38	6.87	0.53
		4	0.79	0.83	0.49	5.49	0.68
**DAB-DETR** **(R50)**	CF_B	1	0.81	0.80	0.52	4.12	0.73
		2	0.76	0.81	0.49	4.37	0.69
		3	0.75	0.77	0.46	4.92	0.66
		4	0.84	0.86	0.53	3.98	0.78
**YOLOv8** **(X6)**	CF_B	1	0.85	0.85	0.51	4.07	0.74
		2	0.82	0.86	0.48	4.22	0.71
		3	0.81	0.83	0.46	4.56	0.68
		4	0.88	0.91	0.54	3.66	0.77
**CMBs-YOLO** **(X6)**	CF_B	1	0.91	0.86	0.58	3.61	0.78
		2	0.86	0.87	0.56	3.77	0.76
		3	0.87	0.84	0.55	4.19	0.73
		4	0.94	0.93	0.63	3.37	0.82

**Figure A2:**
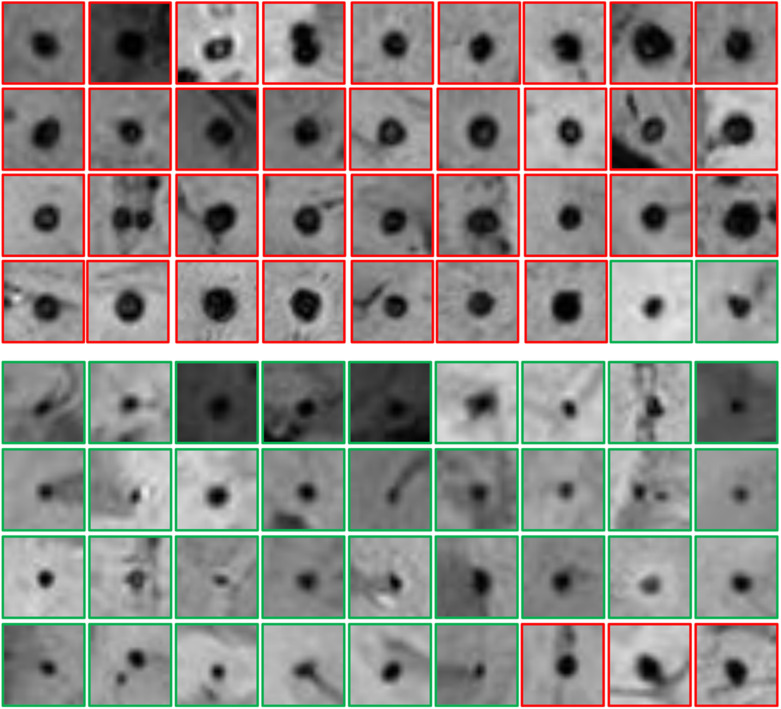
The classification results of CF (B) with text input.

## Data Availability

https://github.com/ai-application/MM-UniCMBs (accessed on 26 September 2024).

## Appendix A

Figure A1 illustrates the detection results from our detection model (CMBs-YOLO-X6). In these visualizations, dashed boxes are color-coded in red, green, and yellow to represent detection scores exceeding 90%, falling between 80 and 90%, and ranging from 70 to 80%, respectively.

## Appendix B

Figure A2 displays the classification results obtained with our CF-B model with text input, across various patients. In this visualization, red boxes are used to identify true CMBs, while green boxes indicate pseudo CMBs. Specifically, in the first block of Figure A2, the green image illustrates instances of false positives as identified with our model. Conversely, the red image in the second block represents cases where the model has missed classifications.

## Appendix C

**Table A1:** The abbreviations and descriptions of detection models.

Abbreviation	Descriptions
R-50 [55]	ResNet with 50 convolution depth
ConvNeXt-T	ConvNext with tiny backbone size, depth = [3, 3, 9, 3]
ConvNeXt-S	ConvNext with small backbone size, depth = [3, 3, 27, 3]
YOLO-N/CMBs-YOLO-N	YOLOv8/CMBs-YOLO methods with N backbone size depth_multiple = 0.33, width_multiple = 0.25, max_channels = 1024
YOLO-S/CMBs-YOLO-S	YOLOv8/CMBs-YOLO methods with S backbone size depth_multiple = 0.33, width_multiple = 0.5, max_channels = 1024
YOLO-M/CMBs-YOLO-M	YOLOv8/CMBs-YOLO methods with M backbone size depth_multiple = 0.67, width_multiple = 0.75, max_channels = 768
YOLO-X6/CMBs-YOLO-X6	YOLOv8/CMBs-YOLO methods with x6 backbone size depth_multiple = 1.0, width_multiple = 1.25, max_channels = 512

**Table A2:** The abbreviations and descriptions of classification models.

Abbreviation	Descriptions
R-18	ResNet with 18 convolution depth
R-50	ResNet with 50 convolution depth
R-101	ResNet with 101 convolution depth
UFv1_XXS	UniFormer version 1 with XXS backbone size depth = [2, 5, 8, 2], embed_dim = [56, 112, 224, 448], head_dim = 28
UFv1_XS	UniFormer version 1 with XS backbone size depth = [3, 5, 9, 3], embed_dim = [64, 128, 256, 512], head_dim = 32
UFv2_B16	UniFormer version 2 with B16 backbone size width = 768, layers = 12, heads = 12, output_dim = 512
UFv2_114	UniFormer version 2 with 114 backbone size width = 1024, layers = 24, heads = 16, output_dim = 768
CF_S w/o text	CMBsFormer with Small backbone size and text inputs (BERT or CLIP) layers = 6, embed_dims = 512, heads = 4, text_channels = 70
CF_B w/o text	CMBsFormer with Base backbone size and text inputs (BERT or CLIP) layers = 12, embed_dims = 768, heads = 8, text_channels = 70
CF_S w/text	CMBsFormer with Small backbone size layers = 6, embed_dims = 512, heads = 4
CF_B w/text	CMBsFormer with Base backbone size layers = 12, embed_dims = 768, heads = 8

## References

[1] Greenberg S.M., Vernooij M.W., Cordonnier C., Viswanathan A., Salman R.A., Warach S., Launer L.J., Buchem M.A.V.B., Breteler M.M.B. (2009). Cerebral microbleeds: A guide to detection and interpretation. Lancet Neurol..

[2] Charidimou A., Werring D.J. (2011). Cerebral microbleeds: Detection, mechanisms and clinical challenges. Future Neurol..

[3] Yamazaki K., Vo-Ho V.K., Bulsara D., Le N. (2022). Spiking neural networks and their applications: A review. Brain Sci..

[4] Dora S., Kasabov N. (2021). Spiking neural networks for computational intelligence: An overview. Big Data Cogn. Comput..

[5] Turkson R.E., Qu H., Mawuli C.B., Eghan M.J. (2021). Classification of Alzheimer’s disease using deep convolutional spiking neural network. Neural Process. Lett..

[6] Ahmadi M., Sharifi A., Hassantabar S., Enayati S. (2021). QAIS-DSNN: Tumor area segmentation of MRI image with optimized quantum matched-filter technique and deep spiking neural network. BioMed Res. Int..

[7] Rajagopal R., Karthick R., Meenalochini P., Kalaichelvi T. (2023). Deep Convolutional Spiking Neural Network optimized with Arithmetic optimization algorithm for lung disease detection using chest X-ray images. Biomed. Signal Process. Control.

[8] Nikseresht G., Agam G., Arfanakis K. (2022). End-to-end task-guided refinement of synthetic images for data efficient cerebral microbleed detection. Proceedings of the 26th International Conference on Pattern Recognition (ICPR).

[9] Ferlin M.A., Grochowski M., Kwasigroch A., Mikołajczyk A., Szurowska E., Grzywińska M., Sabisz A. (2021). A comprehensive analysis of deep neural-based cerebral microbleeds detection system. Electronics.

[10] Ateeq T., Majeed M.N., Anwar S.M., Maqsood M., Rehman Z., Lee J.W., Muhammad K., Wang S.H., Baik S.W., Mehmood I. (2018). Ensemble-classifiers-assisted detection of cerebral microbleeds in brain MRI. Comput. Electr. Eng..

[11] Dou Q., Chen H., Yu L., Zhao L., Qin J., Wang D., Mok V., Shi L., Heng P. (2016). Automatic detection of cerebral microbleeds from MR images via 3D convolutional neural networks. IEEE Trans. Med. Imaging.

[12] Xu C., Wang J., Yang W., Yu H., Yu L., Xia G. (2022). Detecting tiny objects in aerial images: A normalized Wasserstein distance and a new benchmark. ISPRS J. Photogramm. Remote Sens..

[13] Zheng Z., Wang P., Liu W., Li J., Ye R., Ren D. Distance-IoU loss: Faster and better learning for bounding box regression. Proceedings of the AAAI Conference on Artificial Intelligence.

[14] Kaaouana T., Bertrand A., Ouamer F., Ye B.L., Pyatigorskaya N., Bouyahia A., Thiery N., Dufouil C., Delmaire C., Dormont D. (2017). Improved cerebral microbleeds detection using their magnetic signature on T2*-phase-contrast: A comparison study in a clinical setting. NeuroImage Clin..

[15] Hong J., Cheng H., Zhang Y.D., Liu J. (2019). Detecting cerebral microbleeds with transfer learning. Mach. Vis. Appl..

[16] WANG H., Gagnon B. Cerebral microbleed detection by wavelet entropy and naive Bayes classifier. Proceedings of the 2nd International Conference on Biomedical and Biological Engineering 2017 (BBE 2017).

[17] Bao F., Shi M., Macdonald F. Voxelwise detection of cerebral microbleed in CADASIL patients by naïve Bayesian classifier. Proceedings of the 2018 International Conference on Information Technology and Management Engineering (ICITME 2018).

[18] Sundaresan V., Arthofer C., Zamboni G., Dineen R.A., Rothwell P.M., Sotiropoulous S.N., Auer D.P., Tozer D.J., Markus H.S., Miller K.L. (2022). Automated detection of candidate subjects with cerebral microbleeds using machine learning. Front. Neuroinform..

[19] Chesebro A.G., Amarante E., Lao P.J., Meier I.B., Mayeus R., Brickman A.M. (2021). Automated detection of cerebral microbleeds on T2*-weighted MRI. Sci. Rep.

[20] Barnes S.R.S., Haacke E.M., Ayaz M., Boikov A.S., Kirsch W., Kido D. (2011). Semiautomated detection of cerebral microbleeds in magnetic resonance images. Magn. Reson. Imaging.

[21] Dou Q., Chen H., Yu L., Dhi L., Wang D., Mok V., Heng P. (2015). Automatic cerebral microbleeds detection from MR images via independent subspace analysis based hierarchical features. Proceedings of the 2015 37th Annual International Conference of the IEEE Engineering in Medicine and Biology Society (EMBC).

[22] Fazlollahi A., Meriaudeau F., Giancardo L., Villemagne V.L., Rowe C.C., Yates P., Salvado O., Bourgeat P. (2015). Computer-aided detection of cerebral microbleeds in susceptibility-weighted imaging. Comput. Med. Imaging Graph.

[23] Bian W., Hess C.P., Chang S.M., Nelson S.J., Lupo J.M. (2013). Computer-aided detection of radiation-induced cerebral microbleeds on susceptibility-weighted MR images. NeuroImage Clin..

[24] Fazlollahi A., Meriaudeau F., Villemagne V.L., Rowe C.C., Yates P., Salvado O., Bourgeat P. (2014). Efficient machine learning framework for computer-aided detection of cerebral microbleeds using the radon transform. Proceedings of the 2014 IEEE 11th International Symposium on Biomedical Imaging (ISBI).

[25] Morrison M.A., Payabvash S., Chen Y., Avadiappan S., Shah M., Zou X., Hess C.P., Lupo J.M. (2018). A user-guided tool for semi-automated cerebral microbleed detection and volume segmentation: Evaluating vascular injury and data labelling for machine learning. NeuroImage Clin..

[26] Momeni S., Fazlollahi A., Yates P., Rowe C., Gao Y., Liew A., Salvado O. (2021). Synthetic microbleeds generation for classifier training without ground truth. Comput. Methods Programs Biomed..

[27] Liu S., Utriainen D., Chai C., Chen Y., Wang L., Sethi S.K., Xia S., Haacke E.M. (2019). Cerebral microbleed detection using susceptibility weighted imaging and deep learning. Neuroimage.

[28] Wu R., Liu H., Li H., Chen L., Wei L., Huang X., Liu X., Men X., Li X., Ham L. (2023). Deep learning based on susceptibility-weighted MR sequence for detecting cerebral microbleeds and classifying cerebral small vessel disease. BioMedical Eng. Online.

[29] Li T., Zou Y., Bai P., Li S., Wang H., Chen X., Meng Z., Kang Z., Zhou G. (2021). Detecting cerebral microbleeds via deep learning with features enhancement by reusing ground truth. Comput. Methods Programs Biomed..

[30] Zhang Y.D., Hou X.X., Chen Y., Chen H., Yang M., Yang J.Q., Wang S.H. (2018). Voxelwise detection of cerebral microbleed in CADASIL patients by leaky rectified linear unit and early stopping. Multimed. Tools Appl..

[31] Crouzet C., Jeong G., Chae R.H., Lopresti K.T., Dunn C.E., Xie D.F., Agu C., Fang C., Nunes A.C.F., Lau W.L. (2021). Spectroscopic and deep learning-based approaches to identify and quantify cerebral microhemorrhages. Sci. Rep..

[32] Rashid T., Abdulkadir A., Nasrallah I.M., Ware J.B., Liu H.F., Spincemaille P., Romero J.R., Bryan R.N., Heckbert S.R., Habes M. (2021). DEEPMIR: A deep neural network for differential detection of cerebral microbleeds and iron deposits in MRI. Sci. Rep..

[33] Lee H., Kim J.H., Lee S., Jung K.J., Kim W.E., Noh Y., Kim E.Y., Kang K.M., Sohn C.H., Lee D.Y. (2023). Detection of cerebral microbleeds in MR images using a single-stage triplanar ensemble detection network (TPE-Det). J. Magn. Reson. Imaging.

[34] Al-Masni M.A., Kim W.R., Kim E.Y., Noh Y., Kim D.H. (2020). Automated detection of cerebral microbleeds in MR images: A two-stage deep learning approach. NeuroImage Clin..

[35] Fang Z., Zhang R., Guo L., Xia T., Zeng Y., Wu X. (2023). Knowledge-guided 2.5 D CNN for cerebral microbleeds detection. Biomed. Signal Process. Control.

[36] Lu S.Y., Nayak D.R., Wang S.H., Zhang Y.D. (2021). A cerebral microbleed diagnosis method via featurenet and ensembled randomized neural networks. Appl. Soft Comput..

[37] Koschmieder K., Paul M.M., Van den Heuvel T.L.A., Eerden A.V.D., Ginneken B.V., Manniesing R. (2022). Automated detection of cerebral microbleeds via segmentation in susceptibility-weighted images of patients with traumatic brain injury. NeuroImage Clin..

[38] Stanley B.F., Franklin S.W. (2022). Automated cerebral microbleed detection using selective 3D gradient co-occurance matrix and convolutional neural network. Biomed. Signal Process. Control.

[39] Chen Y., Villanueva-Meyer J.E., Morrison M.A., Lupo J.M. (2019). Toward automatic detection of radiation-induced cerebral microbleeds using a 3D deep residual network. J. Digit. Imaging.

[40] Wang S., Tang C., Sun J., Zhang Y. (2019). Cerebral micro-bleeding detection based on densely connected neural network. Front. Neurosci..

[41] Boecking B., Usuyama N., Bannur S., Castro D.C., Schwaighofer A., Hyland S., Wetscherek M., Naumann T., Nori A., Alvarez-Valle J. (2022). Making the most of text semantics to improve biomedical vision–language processing. European Conference on Computer Vision.

[42] Li Z., Li Y., Li Q., Wang P., Guo D., Lu L., Jin D., Zhang Y., Hong Q. (2023). Lvit: Language meets vision transformer in medical image segmentation. IEEE Trans. Med. Imaging.

[43] Liu J., Zhang Y., Chen J.N., Xiao J., Lu Y., Landman B.A., Yuan Y., Yuille A., Tang Y., Zhou Z. Clip-driven universal model for organ segmentation and tumor detection. Proceedings of the IEEE/CVF International Conference on Computer Vision.

[44] Radford A., Kim J.W., Hallacy C., Ramesh A., Goh G., Agarwal S., Sastry G., Askell A., Mishkin P., Clark J. (2021). Learning transferable visual models from natural language supervision. Int. Conf. Mach. Learn. PMLR.

[45] Bhalodia R., Hatamizadeh A., Tam L., Xu Z., Wang X., Turkbey E., Xu D. (2021). Improving pneumonia localization via cross-attention on medical images and reports. Medical Image Computing and Computer Assisted Intervention–MICCAI 2021: 24th International Conference, Strasbourg, France, 27 September–1 October 2021.

[46] Sudre C.H., Van Wijnen K., Dubost F., Adams H., Atkinson D., Barkhof F., Birhanu M.A., Bron E.E., Camarasa R., Chaturvedi N. (2024). Where is VALDO? VAscular Lesions Detection and segmentatiOn challenge at MICCAI 2021. Med. Image Anal..

[47] Ren S., He K., Girshick R., Sun J. (2016). Faster R-CNN: Towards real-time object detection with region proposal networks. IEEE Trans. Pattern Anal. Mach. Intell..

[48] Carion N., Massa F., Synnaeve G., Usunier N., Kirillov A., Zagoruyko S. (2020). End-to-end object detection with transformers. European Conference on Computer Vision.

[49] Ultralytics YOLOv8—Ultralytics Open-Source Research. https://github.com/ultralytics/ultralytics.

[50] Yu T., Li C., Huang J., Xiao X., Zhang X., Li Y., Fu B. (2024). ReF-DDPM: A novel DDPM-based data augmentation method for imbalanced rolling bearing fault diagnosis. Reliab. Eng. Syst. Saf..

[51] Rezatofighi H., Tsoi N., Gwak J.Y., Sadeghian A., Reid I., Savarese S. Generalized intersection over union: A metric and a loss for bounding box regression. Proceedings of the IEEE/CVF Conference on Computer Vision and Pattern Recognition.

[52] Suwalska A., Wang Y., Yuan Z., Jiang Y., Zhu D., Chen J., Cui M., Chen X., Suo C., Polanska J. (2022). CMB-HUNT: Automatic detection of cerebral microbleeds using a deep neural network. Comput. Biol. Med..

[53] Devlin J. (2018). Bert: Pre-training of deep bidirectional transformers for language understanding. arXiv.

[54] Ferlin M., Klawikowska Z., Grochowski M., Grzywinska M., Szurowska E. (2023). Exploring the landscape of automatic cerebral microbleed detection: A comprehensive review of algorithms, current trends, and future challenges. Expert Syst. Appl..

[55] He K., Zhang X., Ren S., Sun J. Deep residual learning for image recognition. Proceedings of the IEEE Conference on Computer Vision and Pattern Recognition.

[56] Lin T. (2017). Focal Loss for Dense Object Detection. arXiv.

[57] Cai Z., Vasconcelos N. (2019). Cascade R-CNN: High quality object detection and instance segmentation. IEEE Trans. Pattern Anal. Mach. Intell..

[58] Duan K., Bai S., Xie L., Qi H., Huang Q., Tian Q. Centernet: Keypoint triplets for object detection. Proceedings of the IEEE/CVF International Conference on Computer Vision.

[59] Zhang H., Chang H., Ma B., Wang N., Chen X. (2020). Dynamic R-CNN: Towards high quality object detection via dynamic training. Computer Vision–ECCV 2020: 16th European Conference, Glasgow, UK, 23–28 August 2020.

[60] Sun P., Zhang R., Jiang Y., Kong T., Xu C.F., Zhan W., Tomizuka M., Li L., Yuan Z.H., Wang C.H. Sparse r-cnn: End-to-end object detection with learnable proposals. Proceedings of the IEEE/CVF Conference on Computer Vision and Pattern Recognition.

[61] Liu Z., Mao H., Wu C.Y., Feichtenhofer C., Darrel T., Xie S. A convnet for the 2020s. Proceedings of the IEEE/CVF Conference on Computer Vision and Pattern Recognition.

[62] Zhu X., Su W., Lu L., Li B., Wang X.G., Dai J. (2020). Deformable detr: Deformable transformers for end-to-end object detection. arXiv.

[63] Guo M.H., Xu T.X., Liu J.J., Liu Z.N., Jiang P.T., Mu T.J., Zhang S.H., Martin R.R., Cheng M.M., Hu S.M. (2022). Attention mechanisms in computer vision: A survey. Comput. Vis. Media.

[64] Zhang H., Li F., Liu S., Zhang L., Su H., Zhu J., Ni L.M., Shum H.Y. (2022). Dino: Detr with improved denoising anchor boxes for end-to-end object detection. arXiv.

[65] Redmon J. (2018). Yolov3: An incremental improvement. arXiv.

[66] Ge Z. (2021). Yolox: Exceeding yolo series in 2021. arXiv.

[67] Sun Z., Ke Q., Rahmani H., Bennamoun M., Wang G., Liu J. (2022). Human action recognition from various data modalities: A review. IEEE Trans. Pattern Anal. Mach. Intell..

[68] Fang Y., Wang W., Xie B., Sun Q., Wu L., Wang X., Huang T., Wang X., Cao Y. Eva: Exploring the limits of masked visual representation learning at scale. Proceedings of the IEEE/CVF Conference on Computer Vision and Pattern Recognition.

[69] Xia P., Hui E.S., Chua B.J., Huang F., Wang Z., Zhang Z., Yu H., Lau K.K., Mak H.K.F., Cao P. (2024). Deep-learning-based MRI microbleeds detection for cerebral small vessel disease on quantitative susceptibility mapping. J. Magn. Reson. Imaging.

[70] Hong J., Wang S.H., Cheng H., Liu J. (2020). Classification of cerebral microbleeds based on fully-optimized convolutional neural network. Multimed. Tools Appl..

[71] Wu X., Hong D., Chanussot J. (2021). Convolutional neural networks for multimodal remote sensing data classification. IEEE Trans. Geosci. Remote Sens..

[72] Shao W., Peng Y., Zu C., Wang M.L., Zhang D.Q. (2020). Hypergraph based multi-task feature selection for multimodal classification of Alzheimer’s disease. Comput. Med. Imaging Graph..

[73] Dai Y., Gao Y., Liu F. (2021). Transmed: Transformers advance multi-modal medical image classification. Diagnostics.

[74] Kurz A., Hauser K., Mehrtens H.A., Henning E.K., Hekler A., Kather J.N., Frohling S., Kalle C.V., Brinker T.J. (2022). Uncertainty estimation in medical image classification: Systematic review. JMIR Med. Inform..

[75] Raj R.J.S., Shobana S.J., Pustokhina I.V., Pustokhin D.A., Gupta D., Shankar K. (2020). Optimal feature selection-based medical image classification using deep learning model in internet of medical things. IEEE Access.

[76] Yang X., Yan J., Ming Q., Wang W., Zhang X., Tian Q. (2021). Rethinking rotated object detection with gaussian wasserstein distance loss. International conference on machine learning. PMLR.

[77] Han Y., Liu X., Sheng Z., Ren Y., Han X., You J., Liu R., Luo Z. Wasserstein loss-based deep object detection. Proceedings of the IEEE/CVF Conference on Computer Vision and Pattern Recognition Workshops.

[78] Diwan T., Anirudh G., Tembhurne J.V. (2023). Object detection using YOLO: Challenges, architectural successors, datasets and applications. Multimed. Tools Appl..

[79] Abdar M., Pourpanah F., Hussain S., Rezazadegan D., Liu L., Ghavamazdeh M., Fieguth P., Cao X.C., Khosravi A., Acharya U.R. (2021). A review of uncertainty quantification in deep learning: Techniques, applications and challenges. Inf. Fusion.

[80] Faghani S., Moassefi M., Rouzrokh P., Khosravi B., Baffour F.I., Ringler M.D., Erickson B.J. (2023). Quantifying uncertainty in deep learning of radiologic images. Radiology.

[81] Zhao R., Wang K., Xiao Y., Gao F., Gao Z. (2024). Leveraging Monte Carlo Dropout for Uncertainty Quantification in Real-Time Object Detection of Autonomous Vehicles. IEEE Access.

[82] Abbaszadeh Shahri A., Shan C., Larsson S. (2022). A novel approach to uncertainty quantification in groundwater table modeling by automated predictive deep learning. Nat. Resour. Res..

[83] Hosseini S.A., Abbaszadeh Shahri A., Asheghi R. (2022). Prediction of bedload transport rate using a block combined network structure. Hydrol. Sci. J..

[84] Abbaszadeh Shahri A., Maghsoudi Moud F. (2021). Landslide susceptibility mapping using hybridized block modular intelligence model. Bull. Eng. Geol. Environ..

[85] Dabov K., Foi A., Katkovnik V., Egiazarian K. (2006). Image denoising with block-matching and 3D filtering. Image processing: Algorithms and systems, neural networks, and machine learning. SPIE.

[86] Naveed K., Abdullah F., Madni H.A., Khan M.A.U., Khan T.M., Naqvi S.S. (2021). Towards automated eye diagnosis: An improved retinal vessel segmentation framework using ensemble block matching 3D filter. Diagnostics.

[87] He Y., Zeng L., Yu W., Gong C. (2020). Noise suppression–guided image filtering for low-SNR CT reconstruction. Med. Biol. Eng. Comput..

